# Patient Safety Culture in Latin American Hospitals: A Systematic Review with Meta-Analysis

**DOI:** 10.3390/ijerph192114380

**Published:** 2022-11-03

**Authors:** Doriam E. Camacho-Rodríguez, Deibys A. Carrasquilla-Baza, Karen A. Dominguez-Cancino, Patrick A. Palmieri

**Affiliations:** 1 Facultad de Enfermería, Universidad Cooperativa de Colombia, Santa Marta 470002, Colombia; 2 EBHC South America: A JBI Affiliated Group, Calle Cartavio 406, Lima 15023, Peru; 3 Addiction Study Program, Université de Sherbrooke, 150, Place Charles-Le Moyne, Bureau 200, Longueuil, QC J4K 0A8, Canada; 4 Escuela de Salud Pública, Universidad de Chile, Av. Independencia 939, Independencia, Santiago de Chile 8380453, Chile; 5 South American Center for Qualitative Research, Universidad Norbert Wiener, Av. Arequipa 444, Lima 15046, Peru; 6 College of Graduate Health Studies, A.T. Still University, 800 West Jefferson Street, Kirksville, MO 63501, USA; 7 Center for Global Nursing, Texas Woman’s University, 6700 Fannin St, Houston, TX 77030, USA

**Keywords:** patient safety, organizational culture, hospitals, leadership, teamwork, communication, staffing, quality improvement, surveys on patient safety culture, SOPS, hospital survey on patient safety culture, HSOPSC, systematic review, Latin America, Spanish

## Abstract

Introduction: Adverse events in hospitals are prevented through risk reduction and reliable processes. Highly reliable hospitals are grounded by a robust patient safety culture with effective communication, leadership, teamwork, error reporting, continuous improvement, and organizational learning. Although hospitals regularly measure their patient safety culture for strengths and weaknesses, there have been no systematic reviews with meta-analyses reported from Latin America. Purpose: Our systematic review aims to produce evidence about the status of patient safety culture in Latin American hospitals from studies using the Hospital Survey on Patient Safety Culture (HSOPSC). Methods: This systematic review was guided by the JBI guidelines for evidence synthesis. Four databases were systematically searched for studies from 2011 to 2021 originating in Latin America. Studies identified for inclusion were assessed for methodological quality and risk of bias. Descriptive and inferential statistics, including meta-analysis for professional subgroups and meta-regression for subgroup effect, were calculated. Results: In total, 30 studies from five countries—Argentina (1), Brazil (22), Colombia (3), Mexico (3), and Peru (1)—were included in the review, with 10,915 participants, consisting primarily of nursing staff (93%). The HSOPSC dimensions most positive for patient safety culture were “organizational learning: continuous improvement” and “teamwork within units”, while the least positive were “nonpunitive response to error” and “staffing”. Overall, there was a low positive perception (48%) of patient safety culture as a global measure (95% CI, 44.53–51.60), and a significant difference was observed for physicians who had a higher positive perception than nurses (59.84; 95% CI, 56.02–63.66). Conclusions: Patient safety culture is a relatively unknown or unmeasured concept in most Latin American countries. Health professional programs need to build patient safety content into curriculums with an emphasis on developing skills in communication, leadership, and teamwork. Despite international accreditation penetration in the region, there were surprisingly few studies from countries with accredited hospitals. Patient safety culture needs to be a priority for hospitals in Latin America through health policies requiring annual assessments to identify weaknesses for quality improvement initiatives.

## 1. Introduction

All human work involves a margin of error [1,2], and health care is no exception [3,4]. Despite years of advancements since the seminal report “To Err Is Human” [5], patient safety continues to be a leading public health problem across countries [6]. Each year, 5.7 to 8.4 million deaths are attributed to poor-quality health services in low- and middle-income countries [7]. Errors in hospitals contribute to at least 15% of hospital costs in the countries of the Organization for Economic Cooperation and Development (OECD), which represents the most economically developed and socially advanced countries in the world [8]. People hospitalized in countries with developing economies are at higher risk for harm from health services because of resource scarcity and the fragility of facilities [9].

The Global Patient Safety Action Plan 2021–2030 urgently recommended that hospitals develop patient safety cultures to reduce the risk of errors and mitigate the harm caused by adverse events [10]. According to Sorra and Dyer [11], patient safety culture refers to the “management and staff values, beliefs, and norms about what is important in a health care organization, how organization members are expected to behave, what attitudes and actions are appropriate and inappropriate, and what processes and procedures are rewarded and punished with regard to patient safety” (p. 1). Creating the conditions for a patient safety culture requires mutual respect, professional collaboration, and the personal support of leaders, clinicians, and staff throughout the care delivery system [12]. Most importantly, the culture needs to shift to a shared mindset that positions patient safety as an important organizational outcome [13]. As such, everyone must be concerned with identifying and reporting latent, or hidden, system errors [14] through nonpunitive error reporting systems [15]. Furthermore, active errors resulting in adverse events should result in a blameless root cause analysis focused on prevention rather than punishment [16]. Resilience to system errors is a quality improvement strategy [17] resulting from an organization-wide concern with failure [18].

At the foundation of a robust patient safety culture is a balance of accountability and patient safety [19]. As a multidimensional organizational attribute linked to quality assurance [20], patient safety cultures positively impact leadership styles, ethical values, communication processes, evidence-based practices, organizational learning, continuous improvement, and person-centered care [21]. Leaders encourage all members of an organization to report latent errors identified from unreliable processes and prepare the organization to manage unexpected system failures caused by active errors [22]. Leaders cultivate positive cultures by affecting the attitudes and behaviors of people, as individuals and units, to embrace patient safety as the normative approach to working with people and caring for patients [23]. With effective leadership and continuous improvement, patient safety becomes a shared organizational property, embedded in the culture, with a collaborative lived experience within units, across departments, and throughout the organization [24]. Hospitals with excellent patient safety cultures have low-risk processes, exhibit highly reliable systems, and produce exceedingly effective outcomes [25].

## 2. Background

Measuring the patient safety culture of hospitals has been an important strategy to identify key areas for continued quality improvement [26] to strengthen patient safety as an organization-wide attribute. Hospital leaders need yearly patient safety culture assessments to learn how to work more effectively with people and where to focus resources to improve system processes, work procedures, and professional practices. Multiple instruments have been developed to measure hospital safety culture, including the Safety Attitudes Questionnaire [27], Safety Organizing Scale [28], and Hospital Survey on Patient Safety Culture (HSOPSC) versions 1.0 [29] and 2.0 [30].

Despite differences in theoretical foundations [31] and conceptual frameworks [32], the instruments similarly focus on measuring seven dimensions of patient safety culture including leadership support, staff attitudes, communication openness, safety systems, incident reporting, organizational learning: continuous improvement, and teamwork [33]. The patient safety culture instrument most often used for research across contexts, cultures, and countries is the HSOPSC [34], which originates from the Surveys on Patient Safety Culture™ program to advance the scientific understanding of patient safety culture in healthcare [35]. Since the release of the HSOPSC 1.0 in 2004, the survey has been translated into 56 languages and dialects for cross-cultural application in more than 100 countries [36]. In addition, studies have been published in journals from at least 32 countries in different regions of the world [37], including China [38], Colombia [39], Croatia [40], Ethiopia [41], Japan [42], Kuwait [33], Norway [43], Peru [44], Spain [45], and others [33,46]. Researchers have also begun to report data from studies using the translated version of the HSOPSC 2.0 [47,48], which was released at the end of 2019 [49].

### 2.1. Hospital Survey on Patient Safety Culture

The HSOPSC 1.0 [50,51] is a 42-item survey with 12 composites, or dimensions, for measuring perceptions about patient safety culture. Each dimension is measured with 3 to 4 items using a 5-point Likert scale with responses ranging from “totally disagree” to “totally agree” or from “never” to “always”. Each dimension and the global measure of patient safety culture can be analyzed at different levels, including individual, unit, department, hospital, and health system. An outcome variable of the survey (overall patient safety grade) is scored as “failing” to “excellent” on a five-point Likert scale [11]. Demographic information provides details about profession (e.g., administrator, nurse, pharmacist, or physician), organization work role (e.g., clerical staff, clinicians, supervisor, manager, or leader), and clinical specialty (e.g., cardiology, critical care, or orthopedics).

The HSOPSC 2.0 [52,53] is a 32-item survey with 10 dimensions. Although HSOPSC 1.0 and 2.0 measure the same areas of patient safety culture, the 2.0 version resulted in substantial changes, including 21 items dropped, 25 items reworded or response options changed, and 10 items added. Early findings suggest that translated versions of the HSOPSC 2.0 will perform better in international hospital environments than original version translations [54]. The HSOPSC 1.0 and 2.0 are available at the Agency for Healthcare Research and Quality resource center for the HSOPSC [49].

The HSOPSC, versions 1.0 [55,56] and 2.0 [30,57], exhibit moderate to strong reliability and validity. For scoring, the HSOPSC item responses can be totaled by dimension as a percentage of positive responses to assess a dimension or to determine the global measure of patient safety culture on a 100-point scale [58]. A dimension is a strength when the aggregate score is equal to or greater than 75% or a weakness when the score is less than 50% [59]. The range between these cut points is considered adequate but requires improvement. Spider diagrams can be used to compare dimensions and composite scores for the HSOPSC 1.0, where perception responses are classified by the percent of positive responses as “excellent” (>90), “good” (70–89), “adequate” (50–69), “fair” (26–49), or ”poor” (<26) [34]. Since the HSOPSC 2.0 scores were observed to be higher than the scores of the HSOPSC 1.0 during pilot studies [60], guidance was provided for version transition, including score interpretation and comparative analysis [61].

### 2.2. Latin America

Spanish language versions of the HSOPSC 1.0 [62] and 2.0 [63] were validated by Spanish-speaking healthcare workers in the United States [50,52]. Although lacking proper survey validation for cross-cultural research, the HSOPSC 1.0 has been also been has been translated, adapted, and validated in Portuguese [64] and Spanish [44] for use in Latin American countries and has been used in studies in Colombia [39], Mexico [65,66], and Peru [67]. These studies reported content validity and item equivalence, establishing language clarity (readability and understandability), and cultural relevance of the HSOPSC 1.0. However, no studies were identified from Latin America using a translated version of the HSOPSC 2.0. An item-level comparison of English and Spanish and versions of the HSOPSC 1.0 from the literature is provided in Supplementary File S1.

Since only one scoping review measured patient safety culture in Brazilian hospitals [68], a systematic review was necessary to produce the evidence for use of the HSOPSC in Latin America before the recommended migration to the HSOPSC 2.0 [49]. For this reason, the current review systematically searched and identified the available evidence for patient safety culture in Latin American hospitals through an analysis of studies that used the HSOPSC. The findings from this study will provide the status of patient safety culture in the region and may help hospitals identify common patient safety culture strengths and weaknesses, compare patient safety culture perceptions with other hospitals in the region, and guide applied research to implement strategies focused on improving patient safety culture. Finally, the findings may inform health policies focused on advancing patient safety culture and guide international accreditation organizations in patient safety culture assessments in Latin American hospitals.

## 3. Methods

This systematic review with meta-analysis was conducted according to the JBI guidelines for evidence synthesis [69]. The results of the study are reported according to the 2020 version of the Preferred Reporting Items for Systematic Reviews and Meta-Analyses (PRISMA) [70]. Further, the study was designed as a living review [71,72] for periodic updates of patient safety culture in Latin America as measured with the HSOPSC.

### 3.1. Review Question

The SPICE (setting, perspective, intervention, comparison, and evaluation) strategy [73,74] was used to compose the review question that guided the search strategy. For the search strategy of the current review, the setting was Latin American hospitals, the perspective was the perception of health care professionals, the interventions were about patient safety culture, the comparison was patient safety culture in relation to organizational culture, and the evaluation was assessed by the HSOPSC measures. Although developing a research question can be complicated for some types of study designs [75,76], the SPICE strategy is flexible for searches that are likely to focus on observational and descriptive study designs [73,74,77]. The SPICE question results in search strategies that are thorough, comprehensive, transparent, and reproducible [78].

### 3.2. Databases

A search of studies was conducted in databases most prominent for research originating from Latin America. These databases included Biblioteca Virtual de Salud (BVS), PubMed, SciELO, and Scopus. In addition, manual searches were conducted from the bibliographic references of included studies, systematic reviews about patient safety, and searches in Google and Google Scholar in English, Portuguese, and Spanish.

### 3.3. Search Strategies

The search strategy included the following MeSH terms and keywords: “patient safety”, “safety culture”, “survey”, “questionnaire”, and “measurement” combined with the word “hospital” in English, Portuguese, and Spanish. These terms and keywords were linked with Boolean operators “and” and “or”. Each search strategy was adapted to the database and language requirements. Consultations were made with a reference librarian to review the search strategy and a linguistics expert for the search term translations. The search strategies were piloted in the databases using the three languages before the study search. The search was limited to Latin American countries, including Mexico. The example search strategies are provided in Supplementary File S2.

### 3.4. Eligibility Criteria

Observational and mixed-methods studies published between January 2011 and December 2021, where patient safety culture was measured with the HSOPSC 1.0 or 2.0 in hospitals located in Latin America, were included in the current review. Studies that did not report measurements for any of the dimensions and conference abstracts, theses, and dissertations were excluded. Narrative, scoping, and systematic reviews related to organization culture or patient safety were noted during the search for a reference list review to identify additional studies meeting the inclusion criteria.

### 3.5. Study Selection

The study selection process was completed sequentially from identification to inclusion according to the PRISMA 2020 flow diagram [79]. First, the documents for review were identified through the database and manual searches. Then, the documents were screened to eliminate duplicates in the reference manager. Next, the documents were added to an Excel spreadsheet for eligibility screening. Two researchers independently reviewed the document titles and abstracts against the inclusion criteria. Potentially relevant documents were retrieved in full-text format and reviewed for inclusion. The rationale for excluding studies was noted for additional review by another researcher. Discrepancies between the paired team of reviewers resulted in documents advancing to the next review round. At the final review round, discrepancies between paired reviewers were reviewed by a third reviewer. There were no unresolved discrepancies.

### 3.6. Data Extraction

Applying an extraction template, study data were independently extracted by two reviewers and organized in an Excel spreadsheet. The minimum information extracted from each study included the publication year, study location (city, country), sample size, sample distribution based on profession, and all data specific to any of the 12 dimensions evaluated by the HSOPSC 1.0 or the 10 dimensions of the HSOPSC 2.0. Reviewers were able to provide additional information during the data extraction by providing a notation about the inclusion rationale. This step provided an opportunity to collect unanticipated data from studies, note data sources requiring additional review, and record unusual or interesting information for later review.

### 3.7. Risk of Bias and Internal Validity

The included studies were assessed for risk of bias using the JBI critical appraisal checklist for analytical cross-sectional studies [80]. The internal validity of each study was assessed with the National Heart, Lung, and Blood Institute quality tool for observational cohorts and cross-sectional studies according to their guideline [81].

### 3.8. Data Synthesis

Data extracted from the studies were organized and analyzed in an Excel spreadsheet. Means and standard deviations were calculated by HSOPSC dimension across studies. The means for the dimensions reported by each study were analyzed, including standard error measurement, followed by a descriptive exploration to assess the sample heterogeneity. Although a random-effects meta-analysis was also completed [82,83], fixed and random-effect analyses were used to compare the results, opting for the most conservative measure [83,84]. Then, a subgroup analysis was performed to account for differences in the sample size and composition [85]. For this analysis, the studies were regrouped into the following three professional categories: nursing teams, physicians, and more than one profession. Finally, a meta-regression was conducted to corroborate the subgroup effects [86]. Inferential statistical analyses were performed with the STATA 16.0 software [87].

### 3.9. Ethical Considerations

Ethics committee approval was not required because the extracted data from the studies were available through searches of publicly accessible databases. However, ethics committee approvals were reported for each study included in this review.

## 4. Results

**Figure 1:**
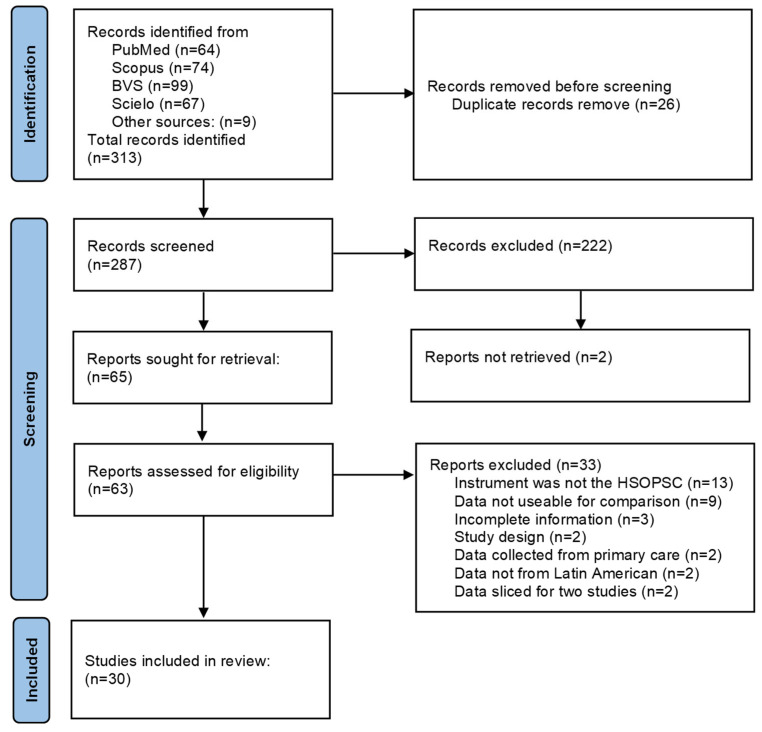
PRISMA 2020 flow diagram for systematic reviews.

After removing duplicate documents, the searches resulted in 313 studies for screening and 63 studies for full-text review. The full screening process resulted in 30 studies included in the meta-analysis as detailed in the PRISMA flowchart in Figure 1.

During the quality assessment, two pairs of studies [88,89,90,91] were found to report analyzed data that were sliced from the same datasets (n = 158, n = 376). As such, the two studies with the more complete data were included for analysis [88,91], and the other two studies were excluded to limit bias [89,90]. Several studies reported small samples with a mixture of clinical and administrative professionals, but the average response rate reported across studies was nearly 80%. Therefore, all 30 studies were determined to be acceptable for meta-analysis.

### 4.1. Study Characteristics

The review included 30 studies from five Latin American countries: 22 studies in Brazil [88,91,92,93,94,95,96,97,98,99,100,101,102,103,104,105,106,107,108,109,110,111], 3 studies in Mexico [112,113,114], 3 studies in Colombia [115,116,117], 1 study in Peru [67], and 1 study in Argentina [118]. All studies reported data from the HSOPSC 1.0; none reported data from the HSOPSC 2.0. The 30 studies totaled 10,915 participants with numbers ranging from 23 participants in a specialized service to 2500 participants in a national population of medical residents. In terms of types of professions, most studies included multiple professionals: 28 studies included nursing staff, 16 studies included physicians and residents, and 13 studies included other professionals and administrative staff. A small number of studies focused exclusively on nurses (7 studies) or physicians (2 studies). The information for studies by country and profession is provided in Table 1.

There were multiple hospitals included in some studies, ranging from one to six. Two studies from Mexico included data from a national [113] (n = 2500) and city [114] (n = 327) population of resident physicians working at different rather than specific hospitals. In general, the data collection process was completed in different venues. For example, most studies (n = 28) were conducted by researchers at hospitals, but one was conducted by a professional association [114] and another during an academic event [67]. In 25 studies, the surveys were delivered physically, but 5 were delivered online [67,95,113,114,117]. The majority of the studies (n = 17) included staff participation from more than one hospital service, including administrative staff and medical residents. In total, one study focused on a high complexity hospital [102], four studies focused on adult [93,99] and neonatal [92,100] intensive care units, five studies focused on perioperative and surgical services [88,101,109,115], and three studies focused on maternity services [103,104,105].

All studies were observational, and most reported cross-sectional designs (n = 29); a single longitudinal study reported three consecutive measurements with averaged results. None of the studies reported interventions as part of the study design. There were some studies (n = 3) with other instruments applied in addition to the HSOPSC 1.0. These instruments included the MISSCARE-Brasil to measure omitted nursing care [107], the Maslach Burnout Inventory to measure burnout, and the Safety Attitudes Questionnaire for construct comparison [93]. Finally, a single study compared the results of hospitals in Brazil and Portugal, but for the current review analysis, only the results from Brazil were extracted [96]. A summary of study characteristics is provided in Supplementary File S3.

### 4.2. HSOPSC 1.0 Dimensions

For measurement of the HSOPSC 1.0 dimensions across studies, the highest percentage of positive responses was observed for “organizational learning: continuous improvement” (68.34%) and “teamwork within units” (67.28%). The consolidated results by study and dimension are provided in Table 2.

The least positive responses were observed for “nonpunitive response to error” (34.8%) and “staffing” (39.5%). By participant profession, the dimension “teamwork within units” was observed to have the most positive score (68.6%) for nurses. The dimensions observed with the least positive scores for nurses were “nonpunitive response to error” (36.5%) and “staffing” (37.1%). For physicians, the dimensions observed to have the least positive scores were “nonpunitive response to error” (27.9%) and “teamwork across hospital units” (37.0%). A detailed summary of the positive responses by dimension is presented for nurses, physicians, and other professionals in Supplementary File S4.

### 4.3. HSOPSC 1.0 Meta-Analysis

**Figure 2:**
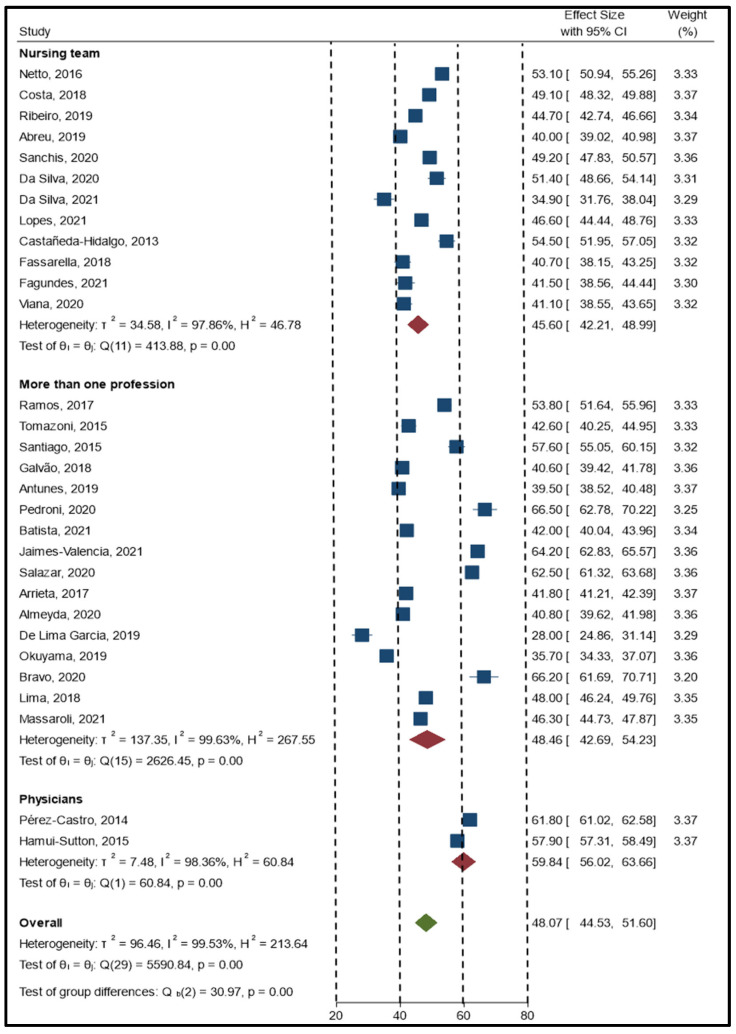
Forest plot of the studies (n = 30) with a positive percent response by professional group.

The meta-analysis resulted in an overall estimator of 48.07 (95% CI, 44.53–51.60), indicating a global perception of patient safety culture that requires improvement. A forest plot of studies with a positive percent response by professional category is provided in Figure 2. The estimator had high heterogeneity with an I^2^ of nearly 98%. For this reason, corroboration for statistically significant differences between groups was calculated.

Nurses were the group with the lowest score for patient safety culture perception with a mean score of 45.60 (95% CI, 42.21–48.99), followed by more than one profession with an average score of 48.46 (95% CI, 42.69–54.23) and physicians with an average score of 59.84 (95% CI, 56.02–63.66). The meta-regression analysis corroborated previous findings by highlighting a statistically significant difference (approximately 14.27 percentage points) between nurses and physicians.

**Figure 3:**
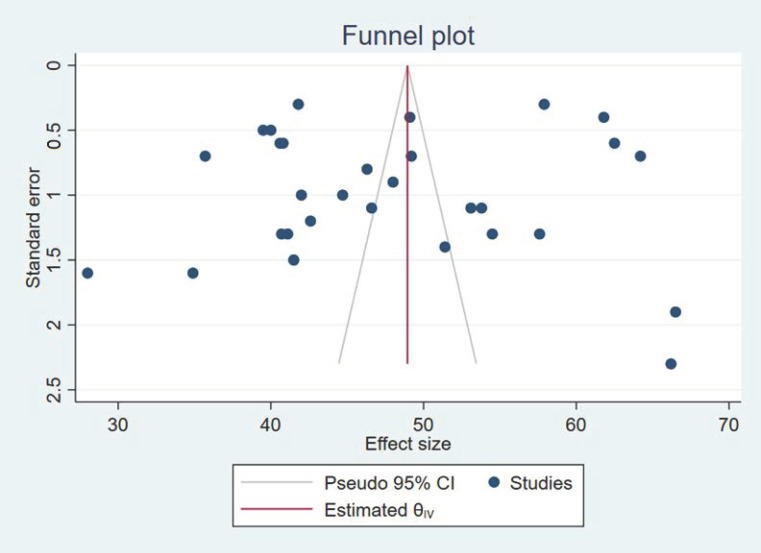
Funnel plot of the studies (n = 30).

Finally, we performed a sensitivity analysis by eliminating studies that were outliers [103,116], but there was no significant change in the estimator. However, publication bias was likely observed because there were no studies with global patient safety culture perceptions of less than 50%. A funnel plot (Figure 3) of the studies confirms the resulting heterogeneity.

## 5. Discussion

The path to achieving patient safety, traveled by many developed countries [119], has not extended too far into Latin America. In the current review, only 30 studies from five countries reported evaluating patient safety culture with the HSOPSC 1.0; no studies used the HSOPSC 2.0. The studies included in this review were nearly all cross-sectional studies measuring the perception of patient safety culture; there were no interventions and few psychometric studies. Developed countries such as Norway, for example, have reported 20 studies using the HSOPSC 1.0, including 7 perception, 6 intervention, and 7 reliability and validity studies [120]. Although not the primary focus of the current review, the studies lacked reporting criteria for the reliability and validity of the translated version of the survey [121,122,123,124]. In the next sections of the discussion, the review findings are presented in terms of the perception of patient safety culture, clinical staffing, teamwork, punitive culture, other measures, and international accreditation. Finally, the limitations section presents several issues realized during this systematic review specific to conducting search strategies in three languages, namely checking citations for multiple analyses of the same dataset and assessing the impact of database selection for Latin American research literature.

### 5.1. Perception of Patient Safety Culture

The general perception of patient safety culture was 48.86% positive responses, which is an indicator that the quality of care should be a priority for the delivery of health services [125]. Spanish researchers reported patient safety culture to be a quality-of-care indicator that benefited the organization as leaders became more involved in the process to prevent risks from becoming repetitive problems [126]. Hospital leaders, managers, supervisors, and coordinators should report a more positive assessment of patient safety than clinicians because of their investment in the organization’s hierarchy and functions. With the advancement of quality improvement initiatives over time, everyone in the organization should report similar levels for the general perception of patient safety culture. If not, then more support and resources are necessary to implement strategies to strengthen patient safety, such as teamwork between units, notification of adverse events, and feedback on errors, to generate organizational learning and continuous improvement [127]. However, there is some contradictory research from intensive care units specific to general versus dimension-level perceptions. For example, intensive care nurses (n = 380) reported high positive perceptions about teamwork within units and organizational learning: continuous improvement, despite having an overall low perception of patient safety culture due to physical and mental demand and overall workload [128]. Importantly, general perceptions need to be carefully interpreted across time within the context, culture, and conditions of the health sector. Finally, general perceptions of patient safety culture may be important human resource indicators as suggested by the relationship between job satisfaction and safety culture reported in a study from Spain [129].

### 5.2. Clinical Staffing

In the current review, statistically significant differences were observed during the meta-analysis for the perception of patient safety culture across the different professional groups. Adequate clinical staffing in a hospital is essential for achieving improved clinical outcomes [130,131,132] and a more favorable patient safety culture [34,58]. The perception of patient safety has been related to the availability of adequate staffing [133]. For example, nurses from countries in different regions of the world, such as Ethiopia [41], Brazil [68], Hungary [134], Iran [135], Spain [136], and Norway [137], reported major concerns about inadequate staffing negatively impacting patient safety and clinical outcomes.

The current study findings about staffing across professions are relevant to the context and culture of the health sector in each country in Latin America. Similar to other studies [34,138,139,140], nurses were more concerned about staffing than physicians. This finding may be explained by most countries having as many or more physicians than nurses per 1000 people, despite nurses being responsible for staffing hospitals for 24 h each day of the year. When comparing similar groups of developed countries, such as members of the OECD, member countries outside Latin America often report having more nurses per capita than physicians. On average there are 8.8 nurses per 1000 population in OECD countries. The Latin American members have fewer nurses (2.6 per 1000 population) than the OECD. Further, the number of nurses per 1000 population in OECD countries ranges from 2.0 nurses in Turkey to 17.7 nurses in Norway. In contrast, there are 3.3 physicians per 1000 population, with a range of 0.5 physicians in Indonesia to 5.4 physicians in Austria. The number of nurses per 1000 population in Latin America varies, but there are more nurses in Costa Rica (3.4) and fewer in Mexico (2.9), Chile (2.7), and Colombia (1.3). When compared with physicians per 1000 population, Turkey (1.9) has an almost equal number of nurses and physicians, while Norway (5.1) has 70% fewer physicians than nurses. Observations from Turkey were similar to Latin American countries. These data are available from the World Bank [141,142,143] and the OECD [144,145,146].

The difference in findings between nurses and other participants in the current review may be related more to the quality of the work environment than the patient safety culture. In a large national hospital study in Chile, Aikens and colleagues [147] reported the nurse workload varied from 6 to 24 patients per nurse across 40 hospitals. Each patient added to the workload of an individual nurse increased mortality (odds ratio 1.04, 95% CI, 1.01–1.07, *p* < 0.01), readmissions (odds ratio 1.02, 95% CI, 1.01–1.03, *p* < 0.01), and length of stay (incident rate ratio 1.04, 95% CI, 1.01–1.06, *p* < 0.05). According to the authors, “Patients in hospitals with 18 patients per nurse, compared with those in hospitals with eight patients per nurse, had 41% higher risk of death, were 20% more likely to be readmitted within 30 days of discharge, had stays that were 41% longer, and were 68% less likely to rate the hospital highly and 55% less likely to recommend the hospital to family and friends” (p. e1151). These findings are consistent with similar large studies from other regions of the world [130,148,149].

The evidence from the current review coupled with the findings from Chile [147] suggests any agenda focused on strengthening patient safety culture requires a better understanding of the working conditions of healthcare professionals, especially nurses [150]. Improvements to the working conditions for nurses may require health policy interventions to establish a minimum staffing standard as an ethical obligation of governments to protect the human rights of patients and to support clinicians in providing safe and high-quality care [151]. Further, research should focus on understanding the relationship between hospital staffing in Latin American countries, such as work conditions and nursing-sensitive qualitative indicators, and patient safety culture.

### 5.3. Teamwork

Similar to studies conducted in other countries [38,120,152], the findings from the current review indicated nurses from Latin American countries perceived higher levels of quality improvement and teamwork contributed to patient safety culture despite poor staffing. The impact of teamwork on patient safety culture probably results from nurses being the core hospital staff with the most direct contact with patients since they are responsible for the continuity of care from diagnostic and therapeutic management to hospital discharge [153]. Nurses working together within units have the most direct impact on care quality and patient safety in hospitals through their continuous quality improvement activities [154] and education that focuses on working in multidisciplinary teams [129].

When performing the meta-analysis for the current review by the professional group, the highest scoring dimension for all groups was “teamwork within units”. Similarly, the teamwork dimension was also the highest scoring for studies conducted in Spain among physicians [155] and other health professions [156]. The quality of teamwork has long been reported to affect the performance of health professionals [157], resulting in organizational outcomes, such as adverse events. From an organizational perspective, the quality of teamwork is impacted by clinical competency, effective care coordination, reliable communication, and seamless collaboration across the organization that extends beyond disciplinary boundaries [158].

Effective teams are better prepared to protect patients from risks and to improve clinical outcomes [159]. In a systematic review investigating the most effective strategies for providing teamwork education programs in hospitals, Eddy et al. [160] recommended six strategies to facilitate programs aligned with cultivating a patient safety culture. The recommendations included suggestions for leaders, such as encouraging clinicians to participate in teamwork education programs, having organizers identify learning needs before implementing a program, providing learning strategies that foster collaboration through debriefing and reflection activities, and using high-fidelity simulation for more realistic opportunities that enhance the communication skills essential for better teamwork. In addition, managers were encouraged to provide opportunities for clinicians to apply their new skills in daily practice.

### 5.4. Punitive Culture

The most concerning perception of nurses in this study was the punitive culture in response to errors. This finding was similar to studies reported from other developing countries [161,162,163]. Since women working in developing countries often experience gender inequality and discrimination, the punitive culture perceived by nurses may result from hospitals being gendered organizations. In this regard, Latin American nurses historically experienced adverse working conditions related to misogynistic attributes, such as machismo culture, paternalistic leaders, and medicalized systems. However, the COVID-19 pandemic resulted in an emerging body of evidence about the widespread abuse, discrimination, harassment, and mistreatment of hospital nurses [164]. Even with greater visibility about the problems female nurses encounter at work, more research is needed to assess the work environment in internationally accredited and non-accredited hospitals in Latin America. However, internationally accredited hospitals should have less gendered cultures and better work environments, resulting in a less punitive culture.

Establishing a nonpunitive culture with open communication is essential to cultivate a robust incident reporting system and facilitating adverse event disclosure [165]. As such, the dimension with the lowest score for physicians was “communication openness”, which suggests a need to strengthen communication [89]. Although more research is necessary, this finding could be related to the punitive culture in some hospitals, where nurses may be less inclined to communicate with physicians or other professionals with positional authority at the hospital. The Healthcare Attribution Error Model [166] was proposed to explain how clinician cynicism, learned helplessness, and organizational inertia result from punitive cultures that silence clinician communication in the context of patient safety and speaking up in the context of error reporting. In a scoping review [167], attribution theory was linked to poor decision-making, errors in judgment, and engagement in two-way communication for speaking-up initiatives with parents of hospitalized children, but there has been no research investigating clinicians speaking up about patient safety concerns. Regardless of the etiology, a nonpunitive response to error reporting and adverse event disclosure is more than a management desire or a hospital policy; instead, a nonpunitive response is an essential tenant for a just culture to improve interpersonal, professional, and institutional capacities for achieving a level of patient safety [168,169] appropriate for the culture and context of Latin America [107].

### 5.5. HSOPSC Studies and Other Measures

There were two studies from Brazil that used another measure in addition to the Portuguese version of the HSOPSC 1.0 [64]. One of these studies [93] applied the HSOPSC 1.0 and the Safety Attitudes Questionnaire and reported a moderate correlation between them (r = 0.66). The other study [107], applied the MISSCARE-Brasil instrument, not the HSOPSC 1.0, to measure omitted nursing care. Although the authors found a more positive patient safety culture was associated with fewer omissions in nursing care, other studies [170] reported that the HSOPSC 1.0 dimensions explained up to 30% of the variance in missed nursing care, 26% of quality-of-care concerns, and 15% of vascular access device events. Further, missed care was significantly associated with falls. Missing nursing care is also proportional to the perceptive lack of patient safety [107] and staffing adequacy [171]. Finally, all lower-scoring dimensions of the HSOPSC 1.0 have correlated to some degree of burnout in other studies [98]. Lower scores for teamwork were most significantly associated with all three dimensions of burnout, including depersonalization, emotional exhaustion, and low professional achievement.

### 5.6. International Accreditation

International hospital accreditation penetration is a useful comparative measurement for health system maturity in developing countries. Specifically, hospital accreditation provides a robust framework for developing the systems, processes, and procedures essential for evidence-based practice, quality improvement, risk management, and patient safety [9,172,173]. However, a recent systematic review (n = 76) described the impact of hospital accreditation on care quality and patient safety as areas requiring more research [174]. Although the review included studies from 22 countries [174], the only country from Latin America was Brazil (n = 9), and few studies (n = 5) addressed organizational culture, including patient safety culture. Because the current literature is focused on understanding the impact of hospital accreditation on organizational culture, the environment of care, and clinical outcomes [175,176,177] (including patient safety culture [174]), we compared the international hospital accreditation penetration in each country of Latin America with the studies included in the current meta-analysis. Brazil reported nearly three-quarters of the hospital-based studies (n = 22) included in this meta-analysis and had nearly five times the total facility accreditations (n = 106) and seven times the hospital accreditations when compared with the next closest country, which was Peru. Despite 18 total accreditations with 12 specifics to hospitals in Peru, there was only one study reported in the literature with data collected at a patient safety conference. There were six other countries with hospital accreditations, but no studies were reported in the literature. The total accreditation activity in Latin America by Accreditation Canada International [178], Acreditas Global [179], Health Facility Accreditation Program [180], and The Joint Commission International [181] is summarized in Table 3.

International hospital accreditation is strongly associated with patient safety culture in other regions of the world [174] and is likely the result of improved systems and more robust processes [182] associated with reporting medication errors [183], disclosing near misses [184], preventing adverse events [185], enhancing organizational learning, and improving teamwork [182]. In a pre- and post-accreditation evaluation of nurses (n = 605) in a Saudi Arabian hospital, a small but statistically significant improvement was observed across all HSOPSC 1.0 dimensions [186]. In a Danish hospital, hospital accreditation was reported to positively impact management priorities that strengthened patient safety culture during accreditation preparation [187]. In South Korea, medication error reporting significantly increased following hospital accreditation [188]. Finally, a recent observational study of nurses reported a weak but statistically significant association between patient safety culture and the hospital accreditation experience [189]. Since compliance with accreditation standards are relatively consistent across the accreditation cycle [190], patient safety culture in internationally accredited healthcare organizations results in sustainable organizational outcomes. For this reason, more research is necessary in Latin America to understand the impact of hospital accreditation on patient safety culture and on other clinical, organizational, and patient outcomes.

### 5.7. Limitations

There are strengths and limitations associated with all systematic reviews. The current review analyzes literature from a world region with multiple developing countries prone to publication bias. The limitations are areas for quality improvement in designing systematic reviews for Latin America. Although the most recognized databases in Latin America were included in the search strategy, national databases were not searched because of resource constraints. Many countries in Latin America maintain national databases for undergraduate and graduate research projects, theses, and dissertations. Some countries require the final research resulting from academic programs to be published in the public domain. Furthermore, professional organizations, scientific societies, and universities commonly publish scientific journals. In the context of Latin America, the research published in these journals is usually captured in the SciELO database. Although theses and dissertations were not included in this review, selection bias was minimized by including the SciELO [191] database in the search strategy. This database provides access to the scientific literature from Latin America published in local, national, and regional journals.

HSOPSC 1.0 studies originating from Latin America often report internal consistency and construct validity but lack power for factor analysis and methodological rigor. For example, a psychometric study reported from Colombia was likely underpowered [192,193] and did not include the expert evaluation of items and dimensions [194,195] or cognitive interviews with bilingual participants to establish content validity and survey equivalence [196,197] before data collection [198,199]. The 12-dimension HSOPSC 1.0 was reported as “not directly applicable to Colombian personnel in a surgical setting”, resulting in the authors recommending a 9-factor, 36-item version. The structural difference can be explained by weak factors consistent with inadequate item wording that caused a high measurement error and a small percentage of common variance [200]. Although the current review did not include a psychometric evaluation, we recognized the potential bias resulting from the HSOPSC 1.0 version selected to measure patient safety culture. For this reason, we recommend increased transparency in reporting the HSOPSC version used for data collection, including source citation and psychometric properties and whether the recommended guidelines for survey translation [201] were followed by the source.

Finally, in the current review, as in others [162,163], a high heterogeneity of studies was observed in the results. Because some studies involved one or several services of an institution, multiple institutions, or the participation of various groups of health professionals, the search strategy was limited to the hospital environment with discernable data related to the research question eligible for data extraction and analysis. All studies included in this review were also evaluated in the databases by the first, corresponding, and last authors and by funding and ethical approval numbers, if available, to identify multiple publications from single data sources. For systematic reviews that include meta-analysis [202], this kind of citation check strategy can minimize the potential for bias from multiple analyses of data from the same database [203] or overlapping portions of data due to salami slicing [203,204,205]. During the full-text assessment of reviewed studies, two sets of studies were found to originate from the same data source [88,89,90,91], which was not disclosed in the subsequent studies despite matching ethical approval numbers.

## 6. Conclusions

Patient safety culture measurement across hospitals in Latin America provides an opportunity to identify common areas for quality improvement to reduce the risk of adverse events and strengthen the overall quality of care. Surprisingly, the literature was limited to studies in only five countries, most of which were in South America and none in Central America. Because patient safety culture may be a relatively unknown or unmeasured concept in most Latin American countries, health professional programs should incorporate more patient safety content into curriculums, with an emphasis on communication, leadership, nonpunitive error reporting, quality improvement, and teamwork.

Despite some penetration of international hospital accreditation in Latin America, there were few studies from countries with accredited hospitals. For this reason, more research should evaluate the impact of accreditation on the patient safety culture in hospitals. Furthermore, quality improvement strategies aligned with advancing patient safety culture in hospitals should be included in the national health policies of Latin American countries. Finally, patient safety culture measurements should be required annually for hospitals and health systems to identify weak dimensions for quality improvement projects and to provide a longitudinal assessment of the organizational culture.

## Figures and Tables

**Table 1:** Characteristics of the reviewed studies (n = 30).

Country (N)	First Author (Year)	Total (N)	Nurses %	Physicians %	Others %
Argentina (1)	Ramos (2017)	191	75.39	12.57	12.04
Brazil (22)	Abreu (2019)	184	50.00	n/a	n/a
	Almeida do Carmo (2020)	301	53.82	37.87	8.31
	Antunes Marques (2019)	514	66.93	15.37	17.70
	Batista (2021)	158	54.43	45.57	n/a
	Campelo (2021)	163	100	n/a	n/a
	Costa (2018)	437	100	n/a	n/a
	de Lima Garcia (2019)	146	80.14	13.70	6.16
	da Silva (2020)	69	100	n/a	n/a
	da Silva (2021)	62	100	n/a	n/a
	Fagundes (2021)	90	100	n/a	n/a
	Fassarella (2018) *	195	100	n/a	n/a
	Galvão (2018)	381	49.87	10.50	39.63
	Lima Andrade (2018)	145	70.34	15.86	13.79
	Massaroli (2021)	291	84.19	n/a	15.81
	Netto (2016)	247	100	n/a	n/a
	Okuyama (2019)	314	45.22	16.88	37.90
	Pedroni (2020)	73	41.10	58.90	n/a
	Ribeiro (2019)	102	100	n/a	n/a
	Sanchis et al., 2020	587	100	n/a	n/a
	Santiago (2015)	88	100	n/a	n/a
	Tomazoni (2014)	141	65.96	34.04	n/a
	Viana (2020)	376	100	n/a	n/a
México (3)	Castañeda-Hidalgo (2013)	195	100	n/a	n/a
	Hamui-Sutton (2015)	2500	n/a	100	n/a
	Pérez-Castro (2014)	327	n/a	100	n/a
Colombia (3)	Bravo (2020)	43	25.58	44.19	30.23
	Jaimes-Valencia (2021)	402	45.77	5.72	48.51
	Salazar (2020)	514	51.56	34.05	14.40
Perú (1)	Arrieta (2018)	1679	17.00	69.00	14.00

* Only the data reported from Brazil were extracted for analysis.

**Table 2:** The positive responses for patient safety culture dimensions by study (n = 30).

First Author (Year)	* D1	D2	D3	D4	D5	D6	D7	D8	D9	D10	D11	D12	Mean	SD
Abreu (2019)	42.2	37.8	58.7	37.3	47.1	32.6	32.3	41.3	34.1	40.6	39.1	37.0	40.0	7.2
Almeida do Carmo (2020)	57.0	57.0	55.0	41.0	35.0	37.0	39.0	31.0	35.0	40.0	43.0	19.0	40.8	11.2
Antunes Marques (2019)	59.4	49.9	49.3	26.4	32.2	33.9	45.3	39.9	37.3	36.2	45.7	18.9	39.5	11.2
Arrieta (2018)	66.0	49.0	61.0	39.0	44.0	37.0	35.0	30.0	43.0	27.0	41.0	30.0	41.8	12.0
Batista (2021)	56.0	56.4	59.5	30.8	45.8	37.8	50.8	45.3	30.7	39.2	32.5	19.3	42.0	12.4
Bravo (2020)	90.3	71.5	75.9	75.0	65.3	65.7	34.3	62.0	73.7	66.0	74.3	40.7	66.2	15.3
Castañeda-Hidalgo (2013)	82.1	63.1	83.6	43.6	44.1	58.0	47.2	67.2	62.6	21.0	45.1	36.9	54.5	18.4
Costa (2018)	57.1	37.4	60.5	47.1	45.3	51.8	36.6	61.4	44.2	36.8	53.1	58.0	49.1	9.2
da Silva (2020)	68.8	42.7	69.6	53.6	51.1	52.2	42.7	40.1	45.0	52.2	33.0	65.8	51.4	11.7
da Silva (2021)	43.1	50.0	56.9	23.6	27.2	31.5	40.0	43.0	31.8	25.9	37.2	9.1	34.9	12.9
de Lima Garcia (2019)	60.8	46.6	21.6	22.3	11.5	9.5	6.8	30.4	46.6	26.5	52.7	0.7	28.0	19.7
Fagundes (2021)	59.4	63.3	62.7	29.4	44.2	38.9	42.9	45.0	26.0	35.5	28.9	21.6	41.5	14.3
Fassarella (2018)	68.0	69.0	57.0	15.0	36.0	39.0	59.0	29.0	23.0	34.0	36.0	23.0	40.7	18.2
Galvão (2018)	58.0	56.0	58.0	35.0	33.0	38.0	41.0	44.0	37.0	33.0	36.0	18.0	40.6	11.9
Hamui-Sutton (2015)	78.1	66.6	76.5	61.0	62.3	60.4	44.7	54.7	62.2	38.3	53.5	36.2	57.9	13.3
Jaimes-Valencia (2021)	85.5	74.2	81.5	69.1	57.4	66.2	38.7	69.1	66.1	55.7	57.2	49.6	64.2	13.3
Lima Andrade (2018)	60.8	66.7	60.0	52.1	31.7	42.1	56.2	39.2	47.8	45.2	37.7	36.1	48.0	11.2
Lopes Campelo (2021)	57.7	58.9	59.5	53.4	42.9	27.6	12.3	56.4	49.7	44.8	41.1	55.2	46.6	14.3
Massaroli (2021)	59.0	58.0	66.0	40.0	41.0	60.0	48.0	53.0	36.0	34.0	44.0	16.0	46.3	14.0
Netto (2016)	73.0	79.0	71.0	61.0	54.0	52.0	53.0	51.0	36.0	45.0	18.0	44.0	53.1	16.9
Okuyama (2019)	51.0	53.0	51.5	23.0	34.7	35.7	40.0	43.8	24.8	28.0	26.8	15.6	35.7	12.4
Pedroni (2020)	78.9	85.5	89.4	83.7	63.0	70.7	72.5	60.0	52.2	43.6	52.1	46.3	66.5	15.9
Pérez-Castro (2014)	70.6	54.3	65.9	64.6	52.0	68.2	51.2	66.9	55.8	67.9	54.1	69.7	61.8	7.6
Ramos (2017)	79.4	65.9	75.0	52.9	48.6	64.4	50.1	59.8	38.0	32.6	41.3	37.9	53.8	15.2
Ribeiro (2019)	50.2	54.1	59.4	43.4	47.8	37.9	47.8	56.9	36.5	32.0	46.2	24.1	44.7	10.5
Salazar (2020)	82.9	70.0	82.9	70.8	56.9	59.7	45.4	70.2	56.2	50.8	57.3	46.3	62.5	12.8
Sanchis (2020)	72.6	66.5	66.3	48.5	46.5	58.8	28.5	59.1	41.2	37.0	43.6	21.9	49.2	15.8
Santiago (2015)	62.1	75.4	74.3	67.8	52.6	54.1	50.4	65.1	50.9	56.2	52.9	29.6	57.6	12.5
Tomazoni (2015)	57.0	61.0	59.0	22.0	36.0	35.0	55.0	47.0	28.0	42.0	51.0	18.0	42.6	14.8
Viana (2020)	68.1	20.7	84.8	21.3	15.7	67.6	50.8	43.6	30.9	21.8	5.6	62.5	41.1	25.4
**Mean Response (M)**	65.2	58.7	65.1	45.1	43.5	47.4	43.3	50.2	42.7	39.6	42.7	33.6	**48.1**	**--**
**Standard Deviation (SD)**	12.3	13.7	13.5	18.4	12.7	15.1	12.8	12.4	12.9	11.7	13.1	17.6	**--**	**9.9**

* Dimensions (D): 1. Teamwork within units; 2. Supervisor expectations and actions to promote patient safety; 3. Organizational learning: continuous improvement; 4. Management support for patient safety; 5. Overall perceptions of patient safety; 6. Feedback and communication about errors; 7. Communication openness; 8. Frequency of events reported; 9. Teamwork across units; 10. Staffing; 11. Handoffs and transitions; and 12. Nonpunitive response to error.

**Table 3:** International accreditation and HSOPSC 1.0 studies (N = 30) by country.

Latin American Countries	Review Studies	Total Accreditations	
		* All	Hospital
**Argentina**	1	3	1
Brazil	22	106	83
Chile	0	2	2
Colombia	3	5	4
Costa Rica	0	3	2
Ecuador	0	3	2
Guatemala	0	2	0
Honduras	0	5	0
Mexico	3	8	7
Nicaragua	0	2	2
Panama	0	3	2
Peru	1	18	12
Totals	30	160	117

* All includes all accreditations, including ambulatory, surgery center, and hospital programs.

## Data Availability

All the data for this study are provided in the figures, tables, and Supplementary Files.

## Supplementary Materials

The following supporting information can be downloaded at: https://www.mdpi.com/article/10.3390/ijerph192114380/s1. Four Supplementary files are provided with the manuscript. Supplementary File S1: Item comparison across English and Spanish versions of the HSOPSC 1.0; Supplementary File S2: Search strategy examples; Supplementary File S3: Characteristics of reviewed studies; Supplementary File S4: Positive responses for safety culture by dimension.

## References

[1] Reason J. (1990). Human Error.

[2] Perrow C. (1984). Normal Accidents: Living with High-Risk Technologies.

[3] Holden R.J. (2009). People or systems? To blame is human. The fix is to engineer. Prof. Saf..

[4] Reason J. (2000). Human error: Models and management. Br. Med. J..

[5] Kohn L.T., Corrigan J.M., Donaldson M.S., Institute of Medicine, Committee on Quality of Health Care in America (2000). To Err is Human: Building a Safer Health System.

[6] Lark M.E., Kirkpatrick K., Chung K.C. (2018). Patient safety movement: History and future directions. J. Hand Surg..

[7] National Academies of Sciences Engineering and Medicine (2018). Crossing the Global Quality Chasm: Improving Health Care Worldwide.

[8] Auraaen A., Slawomirski L., Klazinga N. (2018). The Economics of Patient Safety in Primary and Ambulatory Care.

[9] Wilson R.M., Michel P., Olsen S., Gibberd R.W., Vincent C., El-Assady R., Rasslan O., Qsous S., Macharia W.M., Sahel A. (2012). Patient safety in developing countries: Retrospective estimation of scale and nature of harm to patients in hospital. BMJ.

[10] World Health Organization (2021). Global Patient Safety Action Plan 2021–2030: Towards Eliminating Avoidable Harm in Health Care.

[11] Sorra J.S., Dyer N. (2010). Multilevel psychometric properties of the AHRQ hospital survey on patient safety culture. BMC Health Serv. Res..

[12] Olds D.M., Aiken L.H., Cimiotti J.P., Lake E.T. (2017). Association of nurse work environment and safety climate on patient mortality: A cross-sectional study. Int. J. Nurs. Stud..

[13] Arfanis K., Fioratou E., Smith A. (2011). Safety culture in anaesthesiology: Basic concepts and practical application. Best Pract. Res. Clin. Anaesthesiol..

[14] Petschnig W., Haslinger-Baumann E. (2017). Critical Incident Reporting System (CIRS): A fundamental component of risk management in health care systems to enhance patient safety. Saf. Health.

[15] Feeser V.R., Jackson A.K., Savage N.M., Layng T.A., Senn R.K., Dhindsa H.S., Santen S.A., Hemphill R.R. (2021). When safety event reporting is seen as punitive: “I’ve Been PSN-ed!”. Ann. Emerg. Med..

[16] Peerally M.F., Carr S., Waring J., Dixon-Woods M. (2017). The problem with root cause analysis. BMJ Qual. Saf..

[17] Wagner C., Kristensen S., Sousa P., Panteli D., Busse R., Klazinga N., Panteli D., Quentin W. (2019). Patient safety culture as a quality strategy. Improving Healthcare Quality in Europe: Characteristics, Effectiveness and Implementation of Different Strategies.

[18] Etchegaray J.M., Thomas E.J., Profit J. (2019). Preoccupation with failure and adherence to shared baselines: Measuring high-reliability organizational culture. J. Patient Saf. Risk Manag..

[19] Boysen P.G. (2013). Just culture: A foundation for balanced accountability and patient safety. Ochsner J..

[20] Manzanera R., Moya D., Guilabert M., Plana M., Gálvez G., Ortner J., Mira J.J. (2018). Quality assurance and patient safety measures: A comparative longitudinal analysis. Int. J. Environ. Res. Public Health.

[21] Brittain A.C., Carrington J.M. (2020). Organizational health and patient safety: A systematic review. J. Hosp. Manag. Health Policy.

[22] Singer S.J., Vogus T.J. (2013). Reducing hospital errors: Interventions that build safety culture. Annu. Rev. Public Health.

[23] Barnsteiner J. (2011). Teaching the culture of safety. Online J. Issues Nurs..

[24] Palmieri P.A., DeLucia P.R., Peterson L.T., Ott T.E., Green A., Savage G.T., Ford E.W. (2008). The anatomy and physiology of error in adverse health care events. Patient Safety and Health Care Management.

[25] Lee S.E., Scott L.D. (2018). Hospital nurses’ work environment characteristics and patient safety outcomes: A literature review. West. J. Nurs. Res..

[26] Hughes R.G., Hughes R.G. (2008). Quality methods, benchmarking, and measuring performance. Patient Safety and Quality: An Evidence-Based Handbook for Nurses.

[27] Sexton J.B., Helmreich R.L., Neilands T.B., Rowan K., Vella K., Boyden J., Roberts P.R., Thomas E.J. (2006). The Safety Attitudes Questionnaire: Psychometric properties, benchmarking data, and emerging research. BMC Health Serv. Res..

[28] Vogus T.J., Sutcliffe K.M. (2007). The safety organizing scale: Development and validation of a behavioral measure of safety culture in hospital nursing units. Med. Care.

[29] Nieva V.F., Sorra J. (2003). Safety culture assessment: A tool for improving patient safety in healthcare organizations. Qual. Saf. Health Care.

[30] Sorra J., Zebrak K., Yount N., Famolaro T., Gray L., Franklin M., Smith S.A., Streagle S. (2022). Development and pilot testing of survey items to assess the culture of value and efficiency in hospitals and medical offices. BMJ Qual. Saf..

[31] Halligan M., Zecevic A. (2011). Safety culture in healthcare: A review of concepts, dimensions, measures and progress. BMJ Qual. Saf..

[32] Palmieri P.A., Peterson L.T., Pesta B.J., Flit M.A., Saettone D.M., Fottler M.D., Khatri N., Savage G.T. (2010). Safety culture as a contemporary healthcare construct: Theoretical review, research assessment, and translation to human resource management. Strategic Human Resource Management in Health Care.

[33] Alsalem G., Bowie P., Morrison J. (2018). Assessing safety climate in acute hospital settings: A systematic review of the adequacy of the psychometric properties of survey measurement tools. BMC Health Serv. Res..

[34] Granel-Giménez N., Palmieri P.A., Watson-Badia C.E., Gómez-Ibáñez R., Leyva-Moral J.M., Bernabeu-Tamayo M.D. (2022). Patient safety culture in European hospitals: A comparative mixed methods study. Int. J. Environ. Res. Public Health.

[35] Agency for Healthcare Research and Quality Surveys on Patient Safety Culture™ (SOPS®) Program. https://www.ahrq.gov/sops/about/index.html.

[36] Agency for Healthcare Research and Quality International Use of SOPS. https://www.ahrq.gov/sops/international/index.html.

[37] Waterson P., Carman E.M., Manser T., Hammer A. (2019). Hospital survey on patient safety culture (HSPSC): A systematic review of the psychometric properties of 62 international studies. BMJ Open.

[38] Nie Y., Mao X., Cui H., He S., Li J., Zhang M. (2013). Hospital survey on patient safety culture in China. BMC Health Serv. Res..

[39] Ramírez O.G., Gutiérrez W.A., Vega L.G., Salamanca J.G., Galeano E.M., Gámez A.S. (2011). Cultura de seguridad del paciente por personal de enfermeria en Bogota, Colombia. Cienc. Y Enfermería.

[40] Brborovic H., Sklebar I., Brborovic O., Brumen V., Mustajbegovic J. (2014). Development of a Croatian version of the US Hospital Survey on Patient Safety Culture questionnaire: Dimensionality and psychometric properties. Postgrad. Med. J..

[41] Wami S.D., Demssie A.F., Wassie M.M., Ahmed A.N. (2016). Patient safety culture and associated factors: A quantitative and qualitative study of healthcare workers’ view in Jimma zone Hospitals, Southwest Ethiopia. BMC Health Serv. Res..

[42] Fujita S., Seto K., Kitazawa T., Matsumoto K., Hasegawa T. (2014). Characteristics of unit-level patient safety culture in hospitals in Japan: A cross-sectional study. BMC Health Serv. Res..

[43] Sørskår L.I.K., Abrahamsen E.B., Olsen E., Sollid S.J.M., Abrahamsen H.B. (2018). Psychometric properties of the Norwegian version of the hospital survey on patient safety culture in a prehospital environment. BMC Health Serv. Res..

[44] Palmieri P.A., Leyva-Moral J.M., Camacho-Rodriguez D.E., Granel-Gimenez N., Ford E.W., Mathieson K.M., Leafman J.S. (2020). Hospital survey on patient safety culture (HSOPSC): A multi-method approach for target-language instrument translation, adaptation, and validation to improve the equivalence of meaning for cross-cultural research. BMC Nurs..

[45] Pinheiro M.D.P., Junior O.C.D.S. (2016). Evaluación de la cultura de seguridad del paciente en una organización hospitalaria de un hospital universitario. Enfermería Glob..

[46] Flin R., Burns C., Mearns K., Yule S., Robertson E.M. (2006). Measuring safety climate in health care. Qual. Saf. Health Care.

[47] Lee S.E., Dahinten V.S. (2021). Adaptation and validation of a Korean-language version of the revised hospital survey on patient safety culture (K-HSOPSC 2.0). BMC Nurs..

[48] Suryani L., Letchmi S., Said F.B.M. (2022). Cross-culture adaptation and validation of the Indonesian version of the Hospital Survey on Patient Safety Culture (HSOPSC 2.0). Belitung Nurs. J..

[49] Agency for Healthcare Research and Quality Hospital Survey on Patient Safety Culture. https://www.ahrq.gov/sops/surveys/hospital/index.html.

[50] Sorra J., Gray L., Streagle S., Famolaro T., Yount N., Behm J. (2018). Hospital Survey on Patient Safety Culture: User’s Guide.

[51] Famolaro T., Hare R., Yount N.D., Fan L., Liu H., Sorra J. (2021). Surveys on Patient Safety Culture (SOPS) Hospital Survey 1.0: 2021 User Database Report 21-0016.

[52] Sorra J., Yount N., Famolaro T., Gray L. (2021). Hospital Survey on Patient Safety Culture Version 2.0: User’s Guide, 19(21)-0076.

[53] Famolaro T., Hare R., Yount N.D., Fan L., Liu H., Sorra J. (2021). Surveys on Patient Safety Culture (SOPS) Hospital Survey 2.0: 2021 User Database Report, 21-0017.

[54] Draganović Š., Offermanns G., Davis R.E. (2021). Adaptation of the Agency for Healthcare Research and Quality’s ‘Hospital Survey on Patient Safety Culture’ to the Bosnia and Herzegovina context. BMJ Open.

[55] Blegen M.A., Gearhart S., O’Brien R., Sehgal N.L., Alldredge B.K. (2009). AHRQ’s hospital survey on patient safety culture: Psychometric analyses. J. Patient Saf..

[56] Sorra J., Gray L., Streagle S., Famolaro T., Yount N., Behm J. (2004). Pilot Study: Validity and Reliability of the Hospital Survey on Patient Safety.

[57] Sorra J., Famolaro T., Yount N.D., Caporaso A., Gray L., Zebrak K., Hare R., Thornto S., Fan L., Birch R. (2019). Pilot Test Results from the 2019 AHRQ Surveys on Patient Safety Culture™ (SOPS™) Hospital Survey Version 2.0–Part I: Overall Results, 19-0068.

[58] Danielsson M., Nilsen P., Rutberg H., Årestedt K. (2019). A national study of patient safety culture in hospitals in Sweden. J. Patient Saf..

[59] El-Jardali F., Sheikh F., Garcia N.A., Jamal D., Abdo A. (2014). Patient safety culture in a large teaching hospital in Riyadh: Baseline assessment, comparative analysis and opportunities for improvement. BMC Health Serv. Res..

[60] Sorra J., Famolaro T., Yount N.D. (2019). Transitioning to the SOPS™ Hospital Survey Version 2.0: What’s Different and What to Expect—Part II: Appendixes, 19-0076-1-EF.

[61] Sorra J., Famolaro T., Yount N.D. (2019). Transitioning to the SOPS™ Hospital Survey Version 2.0: What’s Different and What to Expect, Part I: Main Report, 19-0076-1-EF.

[62] Agency for Healthcare Research and Quality (2009). Cuestionario Sobre La De Seguridad De Los Pacientes En Los Hospitales (Version 1.0).

[63] Agency for Healthcare Research and Quality (2019). Cuestionario Sobre La De Seguridad De Los Pacientes En Los Hospitales (Version 2.0).

[64] Fajardo-Dolci G., Rodriguez-Suarez J., Arboleya-Casanova H., Rojano-Fernandez C., Hernandez-Torres F., Santacruz-Varela J. (2010). Cultura sobre seguridad del paciente en profesionales de la salud. Cirugía Y Cir..

[65] Ramirez-Martinez M.E., Gonzalez Pedraza-Aviles A. (2017). Cultura de seguridad y eventos adversos en una clínica de primer nivel. Enfermería Univ..

[66] Arrieta A., Suárez G., Hakim G. (2018). Assessment of patient safety culture in private and public hospitals in Peru. Int. J. Qual. Health Care.

[67] Reis C.T., Laguardia J., Martins M. (2012). Adaptação transcultural da versão brasileira do Hospital Survey on Patient Safety Culture: Etapa inicial. Cad. Saude Publica.

[68] Nascimento Prieto M.M., Pagotti da Fonseca R.E., Zem-Mascarenhas S.H. (2021). Avaliação da cultura de segurança do paciente em hospitais brasileiros através do HSOPSC: Scoping review. Rev. Bras. Enferm..

[69] Aromataris E., Munn Z. (2020). JBI Manual for Evidence Synthesis.

[70] Page M.J., Moher D., Bossuyt P.M., Boutron I., Hoffmann T.C., Mulrow C.D., Shamseer L., Tetzlaff J.M., Akl E.A., Brennan S.E. (2021). PRISMA 2020 explanation and elaboration: Updated guidance and exemplars for reporting systematic reviews. Br. Med. J..

[71] Elliott J.H., Synnot A., Turner T., Simmonds M., Akl E.A., McDonald S., Salanti G., Meerpohl J., MacLehose H., Hilton J. (2017). Living systematic review: 1. Introduction–the why, what, when, and how. J. Clin. Epidemiol..

[72] Kelly S.E., Curran J.A., Tricco A.C. (2022). Managing unmanageable loads of evidence: Are living reviews the answer?. JBI Evid. Synth..

[73] Booth A. (2006). Clear and present questions: Formulating questions for evidence based practice. Libr. Hi Tech.

[74] Booth A., Noyes J., Flemming K., Moore G., Tunçalp Ö., Shakibazadeh E. (2019). Formulating questions to explore complex interventions within qualitative evidence synthesis. BMJ Glob. Health.

[75] Squires J.E., Valentine J.C., Grimshaw J.M. (2013). Systematic reviews of complex interventions: Framing the review question. J. Clin. Epidemiol..

[76] Methley A.M., Campbell S., Chew-Graham C., McNally R., Cheraghi-Sohi S. (2014). PICO, PICOS and SPIDER: A comparison study of specificity and sensitivity in three search tools for qualitative systematic reviews. BMC Health Serv. Res..

[77] Booth A., Facey K.M., Hansen H.P., Single A.N.V. (2017). Qualitative evidence synthesis. Patient Involvement in Health Technology Assessment.

[78] Cooper C., Booth A., Varley-Campbell J., Britten N., Garside R. (2018). Defining the process to literature searching in systematic reviews: A literature review of guidance and supporting studies. BMC Med. Res. Methodol..

[79] Page M.J., McKenzie J.E., Bossuyt P.M., Boutron I., Hoffmann T.C., Mulrow C.D., Shamseer L., Tetzlaff J.M., Akl E.A., Brennan S.E. (2021). The PRISMA 2020 statement: An updated guideline for reporting systematic reviews. Syst. Rev..

[80] Moola S., Munn Z., Tufanaru C., Aromataris E., Sears K., Sfetcu R., Currie M., Qureshi R., Mattis P., Lisy K. (2020). Systematic reviews of etiology and risk. JBI Manual for Evidence Synthesis.

[81] National Heart Lung and Blood Institute Quality Assessment Tool for Observational Cohort and Cross-Sectional Studies. https://www.nhlbi.nih.gov/health-topics/study-quality-assessment-tools.

[82] Brockwell S.E., Gordon I.R. (2001). A comparison of statistical methods for meta-analysis. Stat. Med..

[83] Sheldon T.A., Sutton A.J., Song F., Jones D.R., Abrams K.R. (2000). Methods for Meta-Analysis in Medical Research.

[84] Borenstein M., Hedges L.V., Higgins J.P., Rothstein H.R. (2010). A basic introduction to fixed-effect and random-effects models for meta-analysis. Res. Synth. Methods.

[85] Barker T.H., Migliavaca C.B., Stein C., Colpani V., Falavigna M., Aromataris E., Munn Z. (2021). Conducting proportional meta-analysis in different types of systematic reviews: A guide for synthesisers of evidence. BMC Med. Res. Methodol..

[86] Deeks J.J., Higgins J.P.T., Altman D.G. (2019). Analysing data and undertaking meta-analyses. Cochrane Handbook for Systematic Reviews of Interventions.

[87] StataCorp Stata Statistical Software: Release 16. https://www.stata.com/order/.

[88] Batista J., de Almeida Cruz E.D., Alpendre F.T., da Silva D.P., Brandão M.B., Gabriel C.S. (2021). Diferencias entre los profesionales de enfermería y medicina respecto a la cultura de la seguridad del paciente quirúrgico. Enfermería Glob..

[89] Batista J., de Almeida Cruz E.D., Alpendre F.T., da Silva Stalisz da Paixão D.P., Gaspari A.P., Mauricio A.B. (2019). Cultura de segurança e comunicação sobre erros cirúrgicos na perspectiva da equipe de saúde. Rev. Gauch. Enferm..

[90] Viana K.E., Matsuda L.M., Ferreira A.M.D., dos Reis G.A.X., Souza V.S., Marcon S.S. (2021). Cultura de segurança do paciente na ótica de profissionais de enfermagem. Texto Contexto—Enferm..

[91] Viana K.E., Matsuda L.M., Pereira A.C.S., Oliveira J.L.C., Inoue K.C., Cruz E.D.A. (2020). Cultura de segurança do paciente em hospitais públicos de ensino: Estudo comparativo. Rev. Enferm. UERJ.

[92] Tomazoni A., Rocha P.K.K., de Souza S., Anders J.C., de Malfussi H.C. (2014). Cultura de segurança do paciente em unidades de terapia intensiva neonatal: Perspectivas da equipe de enfermagem e médica. Rev. Lat. Am. Enferm..

[93] Santiago T.H., Turrini R.N. (2015). Cultura e clima organizacional para segurança do paciente em unidades de terapia intensiva. Rev. Esc. Enferm. USP.

[94] Costa D.B., Ramos D., Gabriel C.S. (2018). Cultura de segurança do paciente sob a ótica da equipe de enfermagem em serviços hospitalares, apresentada ao programa de pós-graduação em enfermagem. Texto Contexto Enferm..

[95] Galvão T.F., Lopes M.C.C., Oliva C.C.C., Araújo M.E.A., Silva M.T. (2018). Cultura de segurança do paciente em um hospital universitário. Rev. Lat. Am. Enferm..

[96] Fassarella C.S., Camerini F.G., Henrique D.M., Almeida L.F., Figueiredo M. (2018). Avaliação da cultura de segurança do paciente: Estudo comparativo em hospitais universitários. Rev. Esc. Enferm. USP.

[97] Andrade L.E.L., Lopes J.M., Filho M.C.M.S., Júnior R.F.V., Farias L.P.C.C., dos Santos C.C.M., da Silva Gama Z.A. (2018). Cultura de segurança do paciente em três hospitais brasileiros com diferentes tipos de gestão. Ciência Saúde Coletiva.

[98] De Lima Garcia C., Bezerra I.M.P., Ramos J.L.S., do Valle J.E.T.M.R., de Oliveira M.L.B., Abreu L.C.d. (2019). Association between culture of patient safety and burnout in pediatric hospitals. PLoS ONE.

[99] Ribeiro A.C., Nogueira P.C., Tronchin D.M.R., Rossato V., Serpa L.F. (2019). Cultura de segurança do paciente: Percepção dos enfermeiros em um centro de referência em cardiopneumologia. Texto E Contexto Enferm..

[100] Notaro K.A.M., Corrêa A.R., Tomazoni A., Rocha P.K., Manzo B.F. (2019). Cultura de segurança da equipe multiprofissional em unidades de terapia intensiva neonatal de hospitais públicos. Rev. Lat. Am. Enferm..

[101] Abreu I.M., Rocha R.C., Avelino F., Guimarães D.B.O., Nogueira L.T., Madeira M.Z.A. (2019). Cultura de segurança do paciente em centro cirúrgico: Visão da enfermagem. Rev. Gauch. Enferm..

[102] Sanchis D.Z., Haddad M.C.F.L., Girotto E., Silva A.M.R. (2020). Cultura de segurança do paciente: Percepção de profissionais de enfermagem em instituições de alta complexidade. Rev. Bras. Enferm..

[103] Pedroni V.S., Gouveia H.G., Vieira L.B., Wegner W., Oliveira A.C.S., Santos M.C.D., Carlotto F.D. (2020). Cultura de segurança do paciente na área materno-infantil de hospital universitário. Rev. Gauch. Enferm..

[104] Da Silva P.L., de Oliveira G.M.T., de Brito M.R.L., de Sousa B.B.V., Cardoso R.R., Melo G.T.M. (2020). Cultura de seguridad del paciente en la perspectiva del equipo de enfermería en una maternidad pública. Enfermería Glob..

[105] Do Carmo J.M.A., Mendoza I.Y.Q., Goveia V.R., de Souza K.V., Manzo B.F., de Lima Guimarães G. (2020). Cultura de segurança do paciente em unidades hospitalares de ginecologia e obstetrícia: Estudo transversal. Rev. Bras. Enferm..

[106] Netto F.C.D.B., Severino F.G. (2016). Resultados da avaliação da cultura de segurança em um hospital público de ensino do Ceará. Rev. Bras. Promoção Da Saúde.

[107] Da Silva S.C., Morais B.X., Munhoz O.L., Dal Ongaro J., de Souza Urbanetto J., de Souza Magnago T.S.B. (2021). Cultura de segurança do paciente, cuidados de enfermagem omitidos e suas razões na obstetrícia. Rev. Lat. Am. Enferm..

[108] Campelo C.L., Nunes F.D.O., Silva L.D.C., Guimarães L.F., Sousa S.M.A., de Souza Paiva S. (2021). Cultura de segurança do paciente entre profissionais de enfermagem no ambiente da terapia intensiva. Rev. Esc. Enferm. USP.

[109] Fagundes T.E., Acosta A.S., Gouvea P.B., Massaroli R., Rangel R.C.T., Andrade P.D. (2021). Cultura de segurança do paciente em centro cirúrgico na perspectiva da equipe de enfermagem. J. Nurs. Health.

[110] Massaroli A., De Carli Rodrigues M.E., Kooke K., De Brito Pitilin É., Haag F.B., Araújo J.S. (2021). Avaliação da cultura de segurança do paciente em um hospital do sul do brasil. Cienc. Y Enfermería.

[111] Okuyama J.H.H., Galvão T.F., Crozatti M.T.L., Silva M.T. (2019). Health professionals’ perception of patient safety culture in a university hospital in São Paulo: A cross-sectional study applying the hospital survey on patient safety culture. Sao Paulo Med. J..

[112] Castañeda-Hidalgo H., Hernández R.G., Salinas J.F.G., Zúñiga M.P., Porras G.A., Pérez A.A. (2013). Percepción de la cultura de la seguridad de los pacientes por personal de enfermería. Cienc. Y Enfermería.

[113] Hamui-Sutton A., Pérez-Castro V.J.A., Durán-Pérez V.D., García-Téllez S.E., Fernández-Cantón S.B., Lezana-Fernández M.Á., Carrasco-Rojas J.A. (2015). Percepción de los médicos residentes sobre la cultura de seguridad del paciente en México. Rev. CONAMED.

[114] Perez-Castro J.A., Esparza C.M.J., Martinez L.D.O., Martine L.A.B., Gomez A.A.P., Olvera A.I.G., Bernal M.D., Villarreal H.G.M., Tovar F.R. (2014). Percepción de la cultura de seguridad del paciente en médicos pasantes del servicio social. Rev. CONAMED.

[115] Maya S., Maria A., Marín R., Marcela D. (2020). Cultura de la seguridad del paciente en seis centros quirúrgicos de Antioquia. Rev. Cuid..

[116] Bravo Gómez M., de Pérez L.B.A., Arguello D.K., Moreno X.B., Vega M.P., Naranjo D.J.O., Carvajal R.R. (2020). Cultura de seguridad en profesionales del quirófano en una institución de atención materno infantil. Rev. Cuba. Enfermería.

[117] Jaimes-Valencia M.L., Alvarado-Alvarado A.L., Mejía-Arciniegas C.N., López-Galán A.V., Mancilla-Jiménez V.A., Padilla-García C.I. (2021). Correlación del grado de percepción y cultura de seguridad del paciente en una Institución de tercer nivel 2015–2019. Rev. Cuid..

[118] Ramos F., Coca S.M., Abeldaño R.A. (2017). Percepción de la cultura de seguridad de pacientes en profesionales de una institución argentina. Enfermería Univ..

[119] Longo D.R., Hewett J.E., Ge B., Schubert S. (2005). The long road to patient safety: A status report on patient safety systems. JAMA.

[120] Olsen E., Leonardsen A.-C.L. (2021). Use of the Hospital Survey of Patient Safety Culture in Norwegian hospitals: A systematic review. Int. J. Environ. Res. Public Health.

[121] Wild D., Grove A., Martin M., Eremenco S., McElroy S., Verjee-Lorenz A., Erikson P. (2005). Principles of good practice for the translation and cultural adaptation process for patient-reported outcomes (PRO) measures: Report of the ISPOR task force for translation and cultural adaptation. Value Health.

[122] Eremenco S., Pease S., Mann S., Berry P. (2018). Patient-reported outcome (PRO) consortium translation process: Consensus development of updated best practices. J. Patient-Rep. Outcomes.

[123] Acquadro C., Conway K., Hareendran A., Aaronson N. (2008). Literature review of methods to translate health-related quality of life questionnaires for use in multinational clinical trials. Value Health.

[124] Sousa V.D., Rojjanasrirat W. (2011). Translation, adaptation and validation of instruments or scales for use in cross-cultural health care research: A clear and user-friendly guideline. J. Eval. Clin. Pract..

[125] Zwijnenberg N.C., Hendriks M., Hoogervorst-Schilp J., Wagner C. (2016). Healthcare professionals’ views on feedback of a patient safety culture assessment. BMC Health Serv. Res..

[126] Giménez-Júlvez T., Hernández-García I., Aibar-Remón C., Gutiérrez-Cía I., Febrel-Bordejé M. (2017). Cultura de la seguridad del paciente en directivos y gestores de un servicio de salud. Gac. Sanit..

[127] Lee S.E., Dahinten V.S. (2020). The enabling, enacting, and elaborating factors of safety culture associated with patient safety: A multilevel analysis. J. Nurs. Scholarsh..

[128] Alrabae Y.M.A., Aboshaiqah A.E., Tumala R.B. (2021). The association between self-reported workload and perceptions of patient safety culture: A study of intensive care unit nurses. J. Clin. Nurs..

[129] Merino-Plaza M.J., Carrera-Hueso F.J., Roca-Castello M.R., Morro-Martin M.D., Martinez-Asensi A., Fikri-Benbrahim N. (2018). Relacion entre la satisfaccion laboral y la cultura de seguridad del paciente. Gac. Sanit..

[130] Aiken L.H., Cerón C., Simonetti M., Lake E.T., Galiano A., Garbarini A., Soto P., Bravo D., Smith H.L. (2018). Hospital nurse staffing and patient outcomes. Rev. Médica Clínica Las Condes.

[131] Bruyneel L., Li B., Ausserhofer D., Lesaffre E., Dumitrescu I., Smith H.L., Sloane D.M., Aiken L.H., Sermeus W. (2015). Organization of hospital nursing, provision of nursing care, and patient experiences with care in Europe. Med. Care Res. Rev..

[132] Griffiths P., Ball J., Drennan J., Dall’Ora C., Jones J., Maruotti A., Pope C., Saucedo A.R., Simon M. (2016). Nurse staffing and patient outcomes: Strengths and limitations of the evidence to inform policy and practice. Int. J. Nurs. Stud..

[133] Azyabi A., Karwowski W., Davahli M.R. (2021). Assessing patient safety culture in hospital settings. Int. J. Environ. Res. Public Health.

[134] Granel N., Manresa-Domínguez J.M., Barth A., Papp K., Bernabeu-Tamayo M.D. (2019). Patient safety culture in Hungarian hospitals. Int. J. Health Care Qual. Assur..

[135] Kakemam E., Albelbeisi A.H., Davoodabadi S., Ghafari M., Dehghandar Z., Raeissi P. (2022). Patient safety culture in Iranian teaching hospitals: Baseline assessment, opportunities for improvement and benchmarking. BMC Health Serv. Res..

[136] Granel N., Manresa-Domínguez J.M., Watson C.E., Gómez-Ibáñez R., Bernabeu-Tamayo M.D. (2020). Nurses’ perceptions of patient safety culture: A mixed-methods study. BMC Health Serv. Res..

[137] Jabarkhil A.Q., Tabatabaee S.S., Jamali J., Moghri J. (2021). Assessment of patient safety culture among doctors, nurses, and midwives in a public hospital in Afghanistan. Risk Manag. Healthc. Policy.

[138] Alzahrani N., Jones R., Rizwan A., Abdel-Latif M.E. (2019). Safety attitudes in hospital emergency departments: A systematic review. Int. J. Health Care Qual. Assur..

[139] Verbeek-Van Noord I., Wagner C., Van Dyck C., Twisk J.W.R., De Bruijne M.C. (2013). Is culture associated with patient safety in the emergency department? A study of staff perspectives. Int. J. Qual. Health Care.

[140] Shaw K.N., Ruddy R.M., Olsen C.S., Lillis K.A., Mahajan P.V., Dean J.M., Chamberlain J.M. (2009). Pediatric patient safety in emergency departments: Unit characteristics and staff perceptions. Pediatrics.

[141] The World Bank Physicians (per 1000 People). https://data.worldbank.org/indicator/SH.MED.PHYS.ZS.

[142] The World Bank Number of Surgical Procedures (per 100,000 Population). https://data.worldbank.org/indicator/SH.SGR.PROC.P5.

[143] The World Bank Hospital Beds (per 1000 People). https://data.worldbank.org/indicator/SH.MED.BEDS.ZS.

[144] Organisation for Economic Co-Operation and Development Doctors (Indicator). https://data.oecd.org/healthres/doctors.htm.

[145] Organisation for Economic Co-Operation and Development Nurses (Indicator). https://data.oecd.org/healthres/nurses.htm.

[146] Organisation for Economic Co-Operation and Development Health Spending (Indicator). https://data.oecd.org/healthres/health-spending.htm.

[147] Aiken L.H., Simonetti M., Sloane D.M., Cerón C., Soto P., Bravo D., Galiano A., Behrman J.R., Smith H.L., McHugh M.D. (2021). Hospital nurse staffing and patient outcomes in Chile: A multilevel cross-sectional study. Lancet Glob. Health.

[148] You L.-M., Aiken L.H., Sloane D.M., Liu K., He G.-P., Hu Y., Jiang X.-L., Li X.-H., Li X.-M., Liu H.-P. (2013). Hospital nursing, care quality, and patient satisfaction: Cross-sectional surveys of nurses and patients in hospitals in China and Europe. Int. J. Nurs. Stud..

[149] Aiken L.H., Sloane D.M., Bruyneel L., Van den Heede K., Griffiths P., Busse R., Diomidous M., Kinnunen J., Kózka M., Lesaffre E. (2014). Nurse staffing and education and hospital mortality in nine European countries: A retrospective observational study. Lancet.

[150] Aiken L.H., Sloane D.M., Clarke S., Poghosyan L., Cho E., You L., Finlayson M., Kanai-Pak M., Aungsuroch Y. (2011). Importance of work environments on hospital outcomes in nine countries. Int. J. Qual. Health Care.

[151] Tønnessen S., Scott A., Nortvedt P. (2020). Safe and competent nursing care: An argument for a minimum standard?. Nurs. Ethics.

[152] Skoogh A., Baath C., Hall-Lord M.L. (2022). Healthcare professionals’ perceptions of patient safety culture and teamwork in intrapartum care: A cross-sectional study. BMC Health Serv. Res..

[153] Hernández-Cruz R., Moreno-Monsiváis M.G., Cheverría-Rivera S., Díaz-Oviedo A. (2017). Fatores que influenciam o cuidado de enfermagem omitido em pacientes de um hospital particular. Rev. Lat. Am. Enferm..

[154] Vaismoradi M., Tella S., Logan P., Khakurel J., Vizcaya-Moreno F. (2020). Nurses’ adherence to patient safety principles: A systematic review. Int. J. Environ. Res. Public Health.

[155] Romero M.P., González R.B., Calvo M.S.R. (2017). La cultura de seguridad del paciente en los médicos internos residentes de medicina familiar y comunitaria de Galicia. Atención Primaria.

[156] Laborde M.M., Velázquez M.T.G., Andrés J.M.A., Forner G.R., Rosique A.F.C. (2020). Análisis de la cultura de seguridad del paciente en un hospital universitario. Gac. Sanit..

[157] Firth-Cozens J. (2001). Cultures for improving patient safety through learning: The role of teamwork. Qual. Health Care.

[158] Rosen M.A., DiazGranados D., Dietz A.S., Benishek L.E., Thompson D., Pronovost P.J., Weaver S.J. (2018). Teamwork in healthcare: Key discoveries enabling safer, high-quality care. Am. Psychol..

[159] Lyubovnikova J., West M.A., Dawson J.F., Carter M.R. (2015). 24-Karat or fool’s gold? Consequences of real team and co-acting group membership in healthcare organizations. Eur. J. Work. Organ. Psychol..

[160] Eddy K., Jordan Z., Stephenson M. (2016). Health professionals’ experience of teamwork education in acute hospital settings: A systematic review of qualitative literature. JBI Evid. Synth..

[161] Elmontsri M., Almashrafi A., Banarsee R., Majeed A. (2017). Status of patient safety culture in Arab countries: A systematic review. BMJ Open.

[162] Azami-Aghdash S., Azar F.E., Rezapour A., Azami A., Rasi V., Klvany K. (2015). Patient safety culture in hospitals of Iran: A systematic review and meta-analysis. Med. J. Islam. Repub. Iran.

[163] Okuyama J.H.H., Galvao T.F., Silva M.T. (2018). Healthcare professional’s perception of patient safety measured by the Hospital Survey on Patient Safety Culture: A systematic review and meta-analysis. Sci. World J..

[164] Labrague L.J., De los Santos J.A.A., Fronda D.C. (2021). Perceived COVID-19-associated discrimination, mental health and professional-turnover intention among frontline clinical nurses: The mediating role of resilience. Int. J. Ment. Health Nurs..

[165] Wu A.W., Boyle D.J., Wallace G., Mazor K.M. (2013). Disclosure of adverse events in the United States and Canada: An update, and a proposed framework for improvement. J. Public Health Res..

[166] Palmieri P.A., Peterson L.T., Savage G.T., Fottler M.D. (2009). Attribution theory and healthcare culture: Translational management science contributes a framework to identify the etiology of punitive clinical environments. Biennial Review of Health Care Management: Meso Perspective.

[167] Thirsk L.M., Panchuk J.T., Stahlke S., Hagtvedt R. (2022). Cognitive and implicit biases in nurses’ judgment and decision-making: A scoping review. Int. J. Nurs. Stud..

[168] Sameera V., Bindra A., Rath G.P. (2021). Human errors and their prevention in healthcare. J. Anaesthesiol. Clin. Pharmacol..

[169] The Joint Commission (2018). Developing a reporting culture: Learning from close calls and hazardous conditions. Sentin. Event.

[170] Hessels A.J., Paliwal M., Weaver S.H., Siddiqui D., Wurmser T.A. (2019). Impact of patient safety culture on missed nursing care and adverse patient events. J. Nurs. Care Qual..

[171] Labrague L.J. (2022). Linking nurse practice environment, safety climate and job dimensions to missed nursing care. Int. Nurs. Rev..

[172] Jha A.K. (2018). Accreditation, quality, and making hospital care better. JAMA.

[173] Mansour W., Boyd A., Walshe K. (2020). The development of hospital accreditation in low- and middle-income countries: A literature review. Health Policy Plan..

[174] Hussein M., Pavlova M., Ghalwash M., Groot W. (2021). The impact of hospital accreditation on the quality of healthcare: A systematic literature review. BMC Health Serv. Res..

[175] Lam M.B., Figueroa J.F., Feyman Y., Reimold K.E., Orav E.J., Jha A.K. (2018). Association between patient outcomes and accreditation in US hospitals: Observational study. BMJ.

[176] Desveaux L., Mitchell J.I., Shaw J., Ivers N.M. (2017). Understanding the impact of accreditation on quality in healthcare: A grounded theory approach. Int. J. Qual. Health Care.

[177] Avia I., Hariyati R.T.S. (2019). Impact of hospital accreditation on quality of care: A literature review. Enfermería Clínica.

[178] Accreditation Canada International Internationally Accredited Organizations. https://accreditation.ca/find-intl-accredited-service-provider/.

[179] Acreditas Global Accreditation for international HCPs. https://www.aaahc.org/accreditation/international/.

[180] Health Facility Accreditation Program Search Facilities. https://www.hfap.org/search-facilities/.

[181] The Joint Commission International JCI-Accredited Organizations. https://www.jointcommissioninternational.org/about-jci/accredited-organizations/.

[182] Masciale J.N., Samedy P., Ogden S., Brauer S., Sepkowitz K., Granovsky S. (2018). Is hospital patient safety culture relevant?. J. Clin. Oncol..

[183] Lee H.Y., Lee E.-K. (2020). Safety climate, nursing organizational culture and the intention to report medication errors: A cross-sectional study of hospital nurses. Nurs. Pract. Today.

[184] Toren O., Dokhi M., Ganz F.D. (2021). Hospital nurses’ intention to report near misses, patient safety culture and professional seniority. Int. J. Qual. Health Care.

[185] Yesilyaprak T., Demir Korkmaz F. (2021). The relationship between surgical intensive care unit nurses’ patient safety culture and adverse events. Nurs. Crit. Care.

[186] Al-Awa B., Mazrooa A.A., Rayes O., Hati T.E., Devreux I., Al-Noury K., Habib H., El-Deek B.S. (2012). Benchmarking the post-accreditation patient safety culture at King Abdulaziz University Hospital. Ann. Saudi Med..

[187] Bogh S.B., Blom A., Raben D.C., Braithwaite J., Thude B., Hollnagel E., Plessen C.V. (2018). Hospital accreditation: Staff experiences and perceptions. Int. J. Health Care Qual. Assur..

[188] Lee E. (2016). Safety climate and attitude toward medication error reporting after hospital accreditation in South Korea. Int. J. Qual. Health Care.

[189] Kwan M.R., Seo H.J., Lee S.J. (2021). The association between experience of hospital accreditation and nurses’ perception of patient safety culture in South Korean general hospitals: A cross-sectional study. BMC Nurs..

[190] Devkaran S., O’Farrell P.N. (2014). The impact of hospital accreditation on clinical documentation compliance: A life cycle explanation using interrupted time series analysis. BMJ Open.

[191] Scientific Electronic Library Online About SciELO. https://scielo.org/en/about-scielo.

[192] Nunnally J.C. (1978). Psychometric Theory.

[193] Tabachnick B.G., Fidell L.S. (2001). Using Multivariate Statistics.

[194] Squires A. (2009). Methodological challenges in cross-language qualitative research: A research review. Int. J. Nurs. Stud..

[195] Maneesriwongul W., Dixon J.K. (2004). Instrument translation process: A methods review. J. Adv. Nurs..

[196] Van de Vijver F.J.R., Poortinga Y.H. (1997). Towards an integrated analysis of bias in cross-cultural assessment. Eur. J. Psychol. Assess..

[197] Squires A., Aiken L.H., van den Heede K., Sermeus W., Bruyneel L., Lindqvist R., Schoonhoven L., Stromseng I., Busse R., Brzostek T. (2013). A systematic survey instrument translation process for multi-country, comparative health workforce studies. Int. J. Nurs. Stud..

[198] Erkut S. (2010). Developing multiple language versions of instruments for intercultural research. Child Dev. Perspect..

[199] Mason T.C. (2005). Cross-cultural instrument translation: Assessment, translation, and statistical applications. Am. Ann. Deaf..

[200] Ximénez C. (2016). Recovery of weak factor loadings when adding the mean structure in confirmatory factor analysis: A simulation study. Front. Psychol..

[201] Agency for Healthcare Research and Quality (2022). Translation Guidelines for the AHRQ Surveys on Patient Safety Culture™ (SOPS®).

[202] Hilgard J., Sala G., Boot W.R., Simons D.J. (2019). Overestimation of action-game training effects: Publication bias and salami slicing. Collabra Psychol..

[203] Wallace M.B., Bowman D., Hamilton-Gibbs H., Siersema P.D. (2018). Ethics in publication, part 2: Duplicate publishing, salami slicing, and large retrospective multicenter case series. Endoscopy.

[204] Watson R., Pickler R., Noyes J., Perry L., Roe B., Hayter M., Hueter I. (2015). How many papers can be published from one study?. J. Adv. Nurs..

[205] Tolsgaard M.G., Ellaway R., Woods N., Norman G. (2019). Salami-slicing and plagiarism: How should we respond?. Adv. Health Sci. Educ..

