# Supplementary material for: Patient Safety Culture in Latin American Hospitals: A Systematic Review with Meta-Analysis
Source: Int J Environ Res Public Health. 2022 Nov 3;19(21):14380. doi: 10.3390/ijerph192114380 (PMC9658502; doi:10.3390/ijerph192114380)
Supplement: Supplementary file 1 [file ijerph-19-14380-s001.zip › ijerph-1912595.R2_SUPP-FILE-S2_2022.10.23_FINAL.pdf]

Supplemental File S2. Search Strategy Examples

Database	Search strategy
PUBMED	1. Patient safety (MeSH terms)<br>2. Safety, Patient (All terms)<br>3. 1 OR 2 OR<br>4. Culture (MeSH terms)<br>5. Cultures (All terms)<br>6. 4 OR 5 OR<br>7. Health Care Surveys<br>8. Care Survey, Health<br>9. Care Surveys, Health<br>10. Health Care Survey<br>11. Healthcare Surveys<br>12. Healthcare Survey<br>13. 7 OR 8 OR 9 OR 10 OR 11 OR 12 OR<br>14. Hospitals<br>15. Latin America<br>16. 14 OR 15
Biblioteca Virtual de Salud	(patient safety) AND (culture) AND (fulltext:("1" OR "1" OR "1" OR "1" OR "1" OR "1") AND db:("MEDLINE" OR "LILACS" OR "BDENF") AND type_of_study:("prevalence_studies" OR "incidence_studies") AND la:("en" OR "pt" OR "es") AND pais_assunto:("america do sul" OR "brasil" OR "mexico" OR "colombia" OR "america central" OR "peru" OR "bolivia" OR "costa rica" OR "ecuador" OR "venezuela") AND type:("article")) AND (year_cluster:[2011 TO 2021])<br>(patient safety) AND (health care surveys) AND (fulltext:("1" OR "1") AND mj:("Seguridad del Paciente" OR "Actitud del Personal de Salud") AND type_of_study:("prevalence_studies" OR "incidence_studies" OR "evaluation_studies") AND la:("en" OR "pt" OR "es") AND pais_assunto:("america do sul" OR "brasil" OR "mexico" OR "colombia" OR "argentina" OR "ecuador" OR "peru" OR "venezuela") AND type:("article")) AND (year_cluster:[2011 TO 2021])<br>(patient safety) AND (latin america) AND (fulltext:("1" OR "1") AND mj:("Seguridad del Paciente" OR "Gestión de Riesgos" OR "América Latina") AND type_of_study:("prevalence_studies" OR "incidence_studies" OR "evaluation_studies") AND la:("en" OR "es" OR "pt") AND pais_assunto:("america central" OR "america do sul" OR "chile" OR "colombia" OR "el salvador" OR "guatemala" OR "mexico" OR "brasil" OR "argentina") AND type:("article")) AND (year_cluster:[2011 TO 2021])
Scopus	patient safety and culture and Latin America<br>Filters: Subscribed journals, Research articles, subject areas (Medicine and Dentistry, Business, Nursing and Health Professions), Open access & Open archive<br>patient safety AND hospitals AND Health care surveys<br>Filters: Subscribed journals, Research articles, subject areas (Medicine and Dentistry, Business, Nursing and Health Professions), Open access & Open archive
