# Supplementary material for: Patient Safety Culture in Latin American Hospitals: A Systematic Review with Meta-Analysis
Source: Int J Environ Res Public Health. 2022 Nov 3;19(21):14380. doi: 10.3390/ijerph192114380 (PMC9658502; doi:10.3390/ijerph192114380)
Supplement: Supplementary file 1 [file ijerph-19-14380-s001.zip › ijerph-1912595.R2_SUPP-FILE-S3_2022.10.23_FINAL.pdf]

Supplemental File S3. Characteristics of Reviewed Studies (N=30)

#	First Author (Year)	Services	Hospitals	Data Collection	Nurses	Physicians	Others	Total Participants	Observations
1	Abreu (2019)	Surgical center	1	Paper	92			418	
2	Almeida do Carmo (2020)	Maternal and child hospital	3	Paper	162	114	25	301	
3	Arrieta (2018)	Various units	4	Online	286	1158	235	1679	
4	Batista (2021)	Surgical services	1	Paper	86	72		158	
5	Bravo (2020)	Various units	1	Paper	11	19	13	43	
6	Castañeda-Hidalgo (2013)	Various units	1	Paper	195			195	
7	Costa (2018)	Various units	2	Paper	437			437	
8	da Silva (2020)	Maternal hospital	1	Paper	69			69	
9	da Silva (2021)	Obstetrics units	1	Paper	62			62	Also used MISSCARE-Brazil questionnaire
10	de Lima Garcia (2019)	Pediatric units	1	Paper	117	20	9	146	Also used the Maslach Burnout Inventory
11	Fagundes (2021)	Surgical center	2	Paper	90			90	
12	Fassarella (2018)	Obstetrics, pediatrics, surgical, intensive care units	2	Paper	195			195	The results from Brazil were abstracted
13	Galvão (2018)	Various units	1	Online	190	40	151	381	
14	Hamui-Sutton (2015)	Various units	Various	Online		2500		2500	National survey of physician residents
15	Jaimes-Valencia (2021)	Various units	2	Online	184	23	195	402	The last measurement was taken

#	First Author (Year)	Services	Hospitals	Data Collection	Nurses	Physicians	Others	Total Participants	Observations
16	Lima Andrade (2018)	Various units	3	Paper	102	23	20	145	
17	Lopes Campelo (2021)	Surgical, cardiology, and general intensive care units	1	Paper	163			163	
18	Massaroli (2021)	Various units	1	Paper	245		46	291	
19	Netto (2016)	Various units	1	Paper	247			247	
20	Antunes Marques (2019)	Intensive care units	3	Paper	344	79	91	514	
21	Okuyama (2018)	Various units	1	Paper	142	53	119	314	
22	Pedroni (2020)	Obstetrics and pediatric units	1	Paper	30	43		73	
23	Perez-Castro (2015)	Various units	Various	Online		327		327	National Autonomous University of Mexico
24	Ramos (2017)	Various units	1	Paper	144	24	23	191	
25	Ribeiro (2019)	Units specialized in cardiopulmonary care	1	Paper	102			102	
26	Salazar (2020)	Surgical services	6	Paper	265	175	74	514	
27	Sanchis (2020)	Various units	3	Paper	587			587	
28	Santiago (2015)	Intensive care unit	1	Paper				88	Also used the SAQ questionnaire
29	Tomazoni (2014)	Neonatal intensive care unit	4	Paper	93	48		141	
30	Viana (2020)	Various units	4	Paper	376			376	
