# Supplementary material for: Patient Safety Culture in Latin American Hospitals: A Systematic Review with Meta-Analysis
Source: Int J Environ Res Public Health. 2022 Nov 3;19(21):14380. doi: 10.3390/ijerph192114380 (PMC9658502; doi:10.3390/ijerph192114380)
Supplement: Supplementary file 1 [file ijerph-19-14380-s001.zip › ijerph-1912595.R2_SUPP-FILE-S4_2022.10.24_FINAL.pdf]

Supplemental File S4. Positive Responses for Safety Culture by Dimension

Section A. Positive Nurse Responses for Patient Safety Culture by Dimension

First Author (Year)	D1	D2	D3	D4	D5	D6	D7	D8	D9	D10	D11	D12	Mean	SD
Abreu (2019)	42.2	37.8	58.7	37.3	47.1	32.6	32.3	41.3	34.1	40.6	39.1	37.0	40.0	7.2
Castañeda-Hidalgo (2013)	82.1	63.1	83.6	43.6	44.1	58	47.2	67.2	62.6	21.0	45.1	36.9	54.5	18.4
Costa (2018)	57.1	37.4	60.5	47.1	45.3	51.8	36.6	61.4	44.2	36.8	53.1	58.0	49.1	9.2
da Silva (2020)	68.8	42.7	69.6	53.6	51.1	52.2	42.7	40.1	45.0	52.2	33.0	65.8	51.4	11.7
da Silva (2021)	43.1	50.0	56.9	23.6	27.2	31.5	40.0	43.0	31.8	25.9	37.2	9.1	34.9	12.9
Fagundes (2021)	59.4	63.3	62.7	29.4	44.2	38.9	42.9	45.0	26.0	35.5	28.9	21.6	41.5	14.3
Fassarella (2018)	68.0	69.0	57.0	15.0	36.0	39.0	59.0	29.0	23.0	34.0	36.0	23.0	40.7	18.2
Netto (2016)	73.0	79.0	71.0	61.0	54.0	52.0	53.0	51.0	36.0	45.0	18.0	44.0	53.1	16.9
Lopez Campelo (2021)	57.7	58.9	59.5	53.4	42.9	27.6	12.3	56.4	49.7	44.8	41.1	55.2	46.6	14.3
Ribeiro (2019)	50.2	54.1	59.4	43.4	47.8	37.9	47.8	56.9	36.5	32.0	46.2	24.1	44.7	10.5
Sanchis (2020)	72.6	66.5	66.3	48.5	46.5	58.8	28.5	59.1	41.2	37.0	43.6	21.9	49.2	15.8
Santiago (2015)	62.1	75.4	74.3	67.8	52.6	54.1	50.4	65.1	50.9	56.2	52.9	29.6	57.6	12.5
Viana (2020)	68.1	20.7	84.8	21.3	15.7	67.6	50.8	43.6	30.9	21.8	5.6	62.5	41.1	25.4
**Mean Positive Response**	**61.9**	**55.2**	**66.5**	**41.9**	**42.7**	**46.3**	**41.8**	**50.7**	**39.4**	**37.1**	**36.9**	**37.6**	**42.9**	**--**
**Standard Deviation (SD)**	**11.9**	**16.9**	**9.6**	**15.9**	**10.7**	**12.4**	**12.3**	**11.4**	**11.0**	**10.7**	**13.4**	**18.2**	**--**	**6.7**

Section B. Positive Physician Responses for Patient Safety Culture by Dimension

First Author et al. (Year)	D1	D2	D3	D4	D5	D6	D7	D8	D9	D10	D11	D12	Mean	SD
Hamui-Sutton (2015)	78.1	66.6	76.5	61.0	62.3	60.4	44.7	54.7	62.2	38.3	53.5	36.2	57.9	13.3
Pérez-Castro (2014)	70.6	54.3	65.9	64.6	52.0	68.2	51.2	66.9	55.8	67.9	54.1	69.7	61.8	7.6
**Mean Positive Response**	**74.4**	**60.5**	**71.2**	**62.8**	**57.2**	**64.3**	**48.0**	**60.8**	**59.0**	**53.1**	**53.8**	**53.0**	**59.8**	**--**
**Standard Deviation (SD)**	**5.3**	**8.7**	**7.5**	**2.5**	**7.3**	**5.5**	**4.6**	**8.6**	**4.5**	**20.9**	**0.4**	**23.7**	**--**	**2.8**

Section C. Positive Other Professionals Responses for Patient Safety Culture by Dimension

First Author (Year)	D1	D2	D3	D4	D5	D6	D7	D8	D9	D10	D11	D12	Mean	SD
Almeida do Carmo (2020)	57.0	57.0	55.0	41.0	35.0	37.0	39.0	31.0	35.0	40.0	43.0	19.0	40.8	11.2
Andrade (2018)	60.8	66.7	60.0	52.1	31.7	42.1	56.2	39.2	47.8	45.2	37.7	36.1	48.0	11.2
Arrieta (2017)	66.0	49.0	61.0	39.0	44.0	37.0	35.0	30.0	43.0	27.0	41.0	30.0	41.8	12.0
Antunes Marques (2019)	59.4	49.9	49.3	26.4	32.2	33.9	45.3	39.9	37.3	36.2	45.7	18.9	39.5	11.2
Batista (2021)	56.0	56.4	59.5	30.8	45.8	37.8	50.8	45.3	30.7	39.2	32.5	19.3	42.0	12.4
Bravo (2020)	90.3	71.5	75.9	75.0	65.3	65.7	34.3	62.0	73.7	66.0	74.3	40.7	66.2	15.3
de Lima Garcia (2019)	60.8	46.6	21.6	22.3	11.5	9.5	6.8	30.4	46.6	26.5	52.7	0.7	28.0	19.7
Galvão (2018)	58.0	56.0	58.0	35.0	33.0	38	41.0	44.0	37.0	33.0	36.0	18.0	40.6	11.9
Jaimes-Valencia (2021)	85.5	74.2	81.5	69.1	57.4	66.2	38.7	69.1	66.1	55.7	57.2	49.6	64.2	13.3
Massaroli (2021)	59.0	58.0	66.0	40.0	41.0	60	48.0	53.0	36.0	34.0	44.0	16.0	46.3	14.0
Okuyama (2019)	51.0	53.0	51.5	23.0	34.7	35.7	40.0	43.8	24.8	28.0	26.8	15.6	35.7	12.4
Pedroni (2020)	78.9	85.5	89.4	83.7	63.0	70.7	72.5	60.0	52.2	43.6	52.1	46.3	66.5	15.9
Ramos (2017)	79.4	65.9	75.0	52.9	48.6	64.4	50.1	59.8	38.0	32.6	41.3	37.9	53.8	15.2
Salazar (2020)	82.9	70.0	82.9	70.8	56.9	59.7	45.4	70.2	56.2	50.8	57.3	46.3	62.5	12.9
Tomazoni (2015)	57.0	61.0	59.0	22.0	36.0	35.0	55.0	47.0	28.0	42.0	51.0	18.0	42.6	14.8
**Mean Positive Response**	**66.8**	**61.4**	**63.0**	**45.5**	**42.4**	**46.2**	**43.9**	**48.3**	**43.5**	**40.0**	**46.2**	**27.5**	**47.9**	**--**
**Standard Deviation (SD)**	**12.8**	**10.8**	**16.7**	**20.7**	**14.3**	**17.2**	**14.2**	**13.5**	**13.8**	**11.1**	**11.8**	**14.5**	**--**	**12.0**
